# Flat and complex temperate reefs provide similar support for fish: Evidence for a unimodal species-habitat relationship

**DOI:** 10.1371/journal.pone.0183906

**Published:** 2017-09-05

**Authors:** Avery B. Paxton, Emily A. Pickering, Alyssa M. Adler, J. Christopher Taylor, Charles H. Peterson

**Affiliations:** 1 Institute of Marine Sciences, University of North Carolina at Chapel Hill, Morehead City, North Carolina, United States of America; 2 Department of Biology, University of North Carolina at Chapel Hill, Chapel Hill, North Carolina, United States of America; 3 National Ocean Service, National Centers for Coastal Ocean Science, National Oceanic and Atmospheric Administration, Beaufort, North Carolina, United States of America; Department of Agriculture and Water Resources, AUSTRALIA

## Abstract

Structural complexity, a form of habitat heterogeneity, influences the structure and function of ecological communities, generally supporting increased species density, richness, and diversity. Recent research, however, suggests the most complex habitats may not harbor the highest density of individuals and number of species, especially in areas with elevated human influence. Understanding nuances in relationships between habitat heterogeneity and ecological communities is warranted to guide habitat-focused conservation and management efforts. We conducted fish and structural habitat surveys of thirty warm-temperate reefs on the southeastern US continental shelf to quantify how structural complexity influences fish communities. We found that intermediate complexity maximizes fish abundance on natural and artificial reefs, as well as species richness on natural reefs, challenging the current paradigm that abundance and other fish community metrics increase with increasing complexity. Naturally occurring rocky reefs of flat and complex morphologies supported equivalent abundance, biomass, species richness, and community composition of fishes. For flat and complex morphologies of rocky reefs to receive equal consideration as essential fish habitat (EFH), special attention should be given to detecting pavement type rocky reefs because their ephemeral nature makes them difficult to detect with typical seafloor mapping methods. Artificial reefs of intermediate complexity also maximized fish abundance, but human-made structures composed of low-lying concrete and metal ships differed in community types, with less complex, concrete structures supporting lower numbers of fishes classified largely as demersal species and metal ships protruding into the water column harboring higher numbers of fishes, including more pelagic species. Results of this study are essential to the process of evaluating habitat function provided by different types and shapes of reefs on the seafloor so that all EFH across a wide range of habitat complexity may be accurately identified and properly managed.

## Introduction

Habitat heterogeneity plays an important role in structuring ecological communities, as heterogeneous habitats generally support increased species density, richness, and diversity across terrestrial [1–4], freshwater [5,6], and marine [7,8] ecosystems. Habitat heterogeneity, also referred to as structural complexity, habitat diversity, spatial heterogeneity, architectural complexity, and other variations of these key words [9], influences fundamental processes that organize communities, including species coexistence [10], dispersal [11], recruitment success and mortality [12,13], predation risk [14–16], resource acquisition [15,17,18], and the strength of trophic cascades [19].

Despite the well-documented role of structural complexity in supporting more abundant, more diverse, and richer communities, recent findings challenge the notion that as complexity increases so does the magnitude of community metrics (abundance, diversity, richness), suggesting that under certain scenarios, the relationship between habitat complexity and community metrics is negative or unimodal, rather than positive [9,20]. The ‘area-heterogeneity tradeoff’ combines the conceptual frameworks of niche theory [21] and island biogeography [22–24] to explain why the shape of the relationship between heterogeneity and community metrics may be context dependent [25,26]. The tradeoff hypothesis posits that complex habitats have more fundamental niches and can support more species, yet as heterogeneity increases, the area suitable for each species decreases to the point where the population size decreases and the probability of stochastic extinction increases [25,26]. The applicability of the area-heterogeneity tradeoff, however, has been questioned [27,28], especially as anthropogenic impacts may influence the nature of this relationship [29].

In the marine environment, management decisions to alleviate anthropogenic pressures, such as fishing [30,31], coastal development [32], and tourism [33], often limit human uses of and provide legal protection for habitats characterized by high biodiversity and ecosystem stability [34–37]. Under the assumption that habitats with highest complexity support the most abundant, rich, and diverse concentrations of marine life, habitat-protection decisions commonly prioritize conservation of the most complex habitats as opposed to the least complex habitats [38,39]. This paradigm ignores recent findings and the accompanying conceptual framework (i.e., area-heterogeneity tradeoff), suggesting the most complex habitats, potentially including marine habitats, may not harbor the highest density of individuals and number of species, especially in areas with elevated human influence. Understanding the structure of marine communities as a function of habitat complexity is warranted to ensure that habitat-focused conservation and management efforts encompass appropriate habitat morphologies.

Temperate reefs of the continental shelf of the southeastern United States (US) vary in structural complexity, providing a suitable system to empirically test how to guide habitat-focused management of marine habitats based on structural complexity. These reefs include naturally occurring rocky reefs ranging from flat pavements and rubble fields to substantial ledge systems with up to several meters of vertical relief [40,41]. The continental shelf also forms the resting place for shipwrecks [42], as well as architecturally unique human-made structures, ranging from concrete pipes to large ships intentionally sunk to enhance fisheries [42–44]. While these natural and artificial reefs vary in morphology, they also experience dramatic state changes due to sedimentary, biological, and physical processes that alter the degree of sediment cover by alternately burying and exposing the flattest reefs [40,41,45–47].

Temperate reefs, including flat-to-complex rocky reefs and artificial reefs, of the southeastern US are federally-designated essential fish habitat (EFH) under the Magnuson-Stevens Fishery Conservation and Management Act (2007) because they function as nurseries, refugia, foraging sites, and spawning grounds. Unlike rocky reefs and artificial reefs, shipwrecks are not designated as EFH, despite forming important habitat for fishes. Rocky reefs, artificial reefs, and shipwrecks provide habitat for a diversity of fishes, ranging from tropical and subtropical to warm-temperate reef fishes and coastal pelagics. Temperate reefs also support fishes in the federally-managed snapper-grouper complex [48,49] whose status is of particular concern because of their recreational and commercial value and their frequently depressed numbers [50–52]. These reefs are valuable for the coastal economy and culture because they create and sustain commercially and recreationally important fisheries and recreational diving opportunities. Aside from risks of overexploitation through fisheries, emerging risks on the continental shelf from offshore renewable and conventional energy development makes understanding the habitat function of these reefs pressing.

The objectives of this study were to: 1) Quantify how structural complexity of temperate reefs, measured as reef rugosity, influences fish communities; and 2) Provide management and conservation recommendations based on habitat complexity to achieve goals of fisheries and ecosystem management. This study is essential to the process of evaluating habitat function provided by different types and shapes of hard structures on the seafloor so that EFH may be accurately identified and effectively managed.

## Materials and methods

### Survey sites

**Fig 1:**
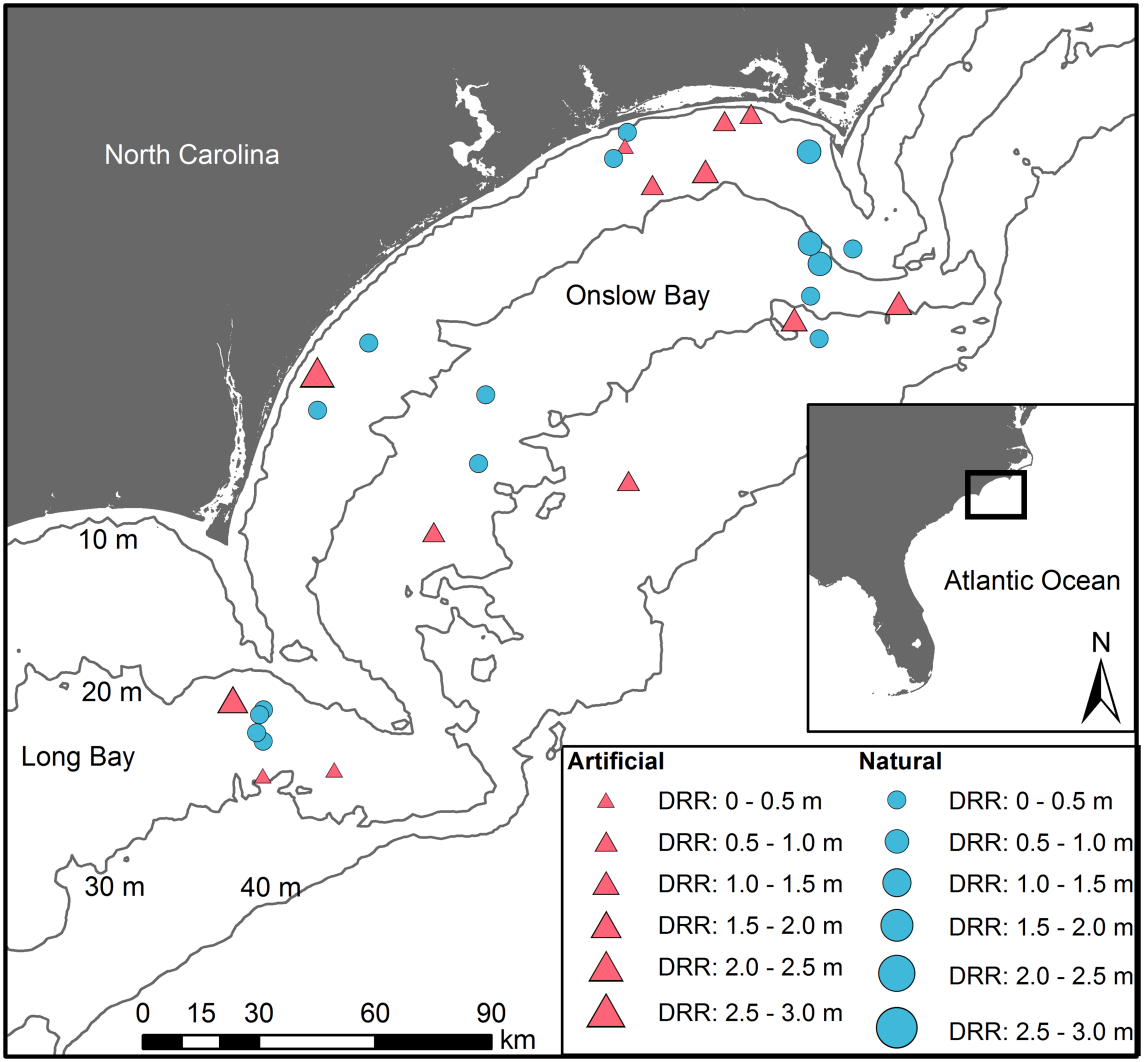
Thirty temperate reefs, including natural (blue circles) and artificial (red triangles) reefs, surveyed on the continental shelf of NC. Point size is proportional to mean digital reef rugosity (DRR) from transects on the particular reef. Symbols overlap for two artificial reefs located in northern Onslow Bay.

We conducted scuba-diver surveys of thirty reefs off the coast of North Carolina (NC) along the southeastern US continental shelf (Fig 1; S1 Table). We selected these thirty reefs, including sites representative of different topographic complexities. Twenty-three of these warm-temperate reefs occur within Onslow Bay, NC, whereas the remaining seven sites lie in northeastern Long Bay, NC within an area designated for potential offshore wind energy development. Sites in Onslow Bay were selected *a priori* based on a design that was stratified by water depth, which is correlated with distance from shore. Sites in Long Bay were selected from side-scan sonar and multibeam bathymetry datasets acquired during a seafloor mapping cruise in June 2013 [53]. Sixteen of the thirty sites are natural reefs, ranging from flat pavements to ledges, and fourteen are artificial, human-made reefs include historic shipwrecks, as well as concrete pipes and ships purposely sunk as part of the NC Artificial Reef Program.

Sites were sampled seasonally during 2013–2015 (S1 Table). Most sites were sampled during each season (e.g., summer, fall, etc.), but due to rough sea conditions, several sites were sampled during only one season (S1 Table). At each site, two 30-m long transects were established along prominent reef features. When no prominent feature existed, the transect direction was selected from a list of randomly generated compass headings. The transect location at each site varied among seasons. Diver surveys to quantify fishes and structural complexity were conducted along each transect. No specific permissions were required to survey the selected thirty reefs.

### Fish community assessments

To quantify fish community metrics, such as abundance and composition, divers sampled along a 30-m x 4-m (120-m^2^) belt transect [54–56], while recording the species and abundance of all fishes present throughout the water column, including both conspicuous and cryptic categories of reef fishes, to the lowest taxonomic level possible. Fish length was estimated to the nearest cm. Biomass was calculated with the length-weight power function as:

$$W=aL^{b}$$

where *L* is length (cm) recorded during the fish transect and *W* is weight (g). When there was a school of fish that spanned different sizes, *L* was calculated as the midpoint of the recorded size range. Species-specific morphometric values for *a* and *b* were obtained from Fishbase [57]. For species that were identifiable only to the family level, the average morphometric values for other known species in the family observed on the reefs were used. Weight was converted to kg. When two belt transects were conducted at a reef during a single sampling season, fish abundances and biomasses from each transect were averaged as a single sample to calculate respective abundance and biomass metrics. We computed species richness (S) at the finest taxonomic resolution possible (e.g., species), as well as for families. In addition to overall metrics that were inclusive of all sizes of fishes, size-class specific metrics were calculated for small fishes 1–10 cm, medium fishes 11–29 cm, large fishes 30–49 cm, and extra-large fishes *≥* 50 cm. Ethics approval was not required, as this was an observational study where fishes were visually counted and identified *in situ* by scientific divers.

### Structural complexity

To document how structural complexity affects fish use of temperate reefs, we collected measurements of the contour of each reef using an Onset HOBO U20 Titanium Water Level Logger (U20-001-02-Ti) containing a pressure-transducer that records pressure at 1 Hz, from which bottom elevations are inferred. As per methods in Dustan et al. [8], a diver swam over the reef with the logger suspended from a line and positioned as close to the substrate as possible. If benthic organisms, such as sponges, coral, and dense meadows of macroalgae, rose above the substrate preventing divers from positioning the logger close to the substrate, then divers moved the logger above these habitat-forming animals and plants to avoid damaging them and to account for the contributions of these organisms to reef complexity. The logger was moved at ~ 10 cm per second over the length of each 30-m transect. The logger was raised 1 m above and rapidly lowered back down to the substrate surface in a spike motion five times at the start of each transect, three times every 5 m thereafter, and five times at the end of each transect. Since the logger records continuously, these spikes were used to identify each transect within the data record and convert sample time to distance along transects. During post-dive processing, the distance calibration spikes were removed from each file, and raw pressures recorded by the pressure-transducer were converted from units of PSI to m, assuming an atmospheric pressure of 1 atm. If the diver swim-speed differed from the target rate of ~ 10 cm per second, then the actual swim speed was computed from the transect length and time between calibration spikes and used to determine distance along the 30-m transect.

For each transect, reef shape was visualized by plotting water depth against along-transect distance. Mean, minimum, and maximum depths were determined for each transect. Vertical relief of each transect was computed as the difference between the minimum and maximum depth. Digital reef rugosity (DRR) [8] was calculated as the standard deviation of depths along each transect (m). An alternative measure of rugosity was calculated as the ratio of the actual surface contour distance to the linear transect distance as:

$$C=\frac{D}{L}$$

where *C* = rugosity, *L* = linear distance of transect (m), and *D* = distance of transect following the natural reef surface contour (m) [7,58]. Distance of the natural surface contour (*D*) was computed as the sum of the hypotenuses between every two successive depth measurements recorded by the water level logger. We compared the two values for rugosity, DRR and C, and the one value for vertical relief, to ensure that these metrics were correlated across transects, and upon confirmation, DRR was selected as the metric of choice because of its precision and previously documented positive correlation with fish diversity on coral reefs [8]. To visualize the distribution of complexity values across reefs, kernel density [59] was estimated using the ‘stats’ package in R [60].

Spatial variability of each structural complexity transect was visualized with variograms. Variograms decompose the spatial variability in a transect among distance classes [61,62]. In the case of the structural complexity transects, distance classes corresponded to every measurement of depth (m) separated by 10 cm through to 300 cm (30 m), or the entire transect distance (e.g, 10 cm, 20 cm, 30 cm… 280 cm, 290 cm, 300 cm). The variance attributed to each of these distance classes is called the semivariance. Semivariance was calculated as:

$$\gamma \left(d\right)=\;\frac{1}{2N\left(d\right)}\sum_{i=1}^{W\left(d\right)}{\left(y_{i}-y_{i+d}\right)}^{2}$$

where *γ(d)* is the semivariance at distance class *d*, *N(d)* is the number of pairs for separation of distance class *d*, *y*_*i*_ is the depth at location *i* and *y*_*i+d*_ is the depth at location *i* plus the distance class value *d*, and *W(d)* is the final location of the transect that corresponds to distance class *d* [62,63]. Semivariance was plotted against distance classes. We plotted semivariance up to distance classes that were half the transect length to ensure that we plotted the spatially structured component of each transect [62]. Resulting variograms depicted the spatial scale over which the complexity of each reef varied.

### Water temperature

We measured temperature on each transect using the same Onset HOBO U20 Titanium Water Level Logger (U20-001-02-Ti) that we used to measure structural complexity. The water level logger recorded temperature every second over the duration of each transect. These raw temperature values were used to calculate mean temperature (°C) over each transect. When multiple transects were conducted in the same sampling season, water temperatures were averaged as a single replicate.

### Sediment cover

We measured sediment depth using a hollow 2 cm diameter PVC rod containing graduated markings to the nearest cm. The rod was shaped as a ‘T,’ so that divers could apply pressure on the top, horizontal component of the ‘T’ to press the rod into the sediment. Sediment depth measurements were obtained every three meters along the same transect that fishes and structural complexity were sampled. Sediment data were also averaged over multiple transects when a reef was surveyed more than once in a sampling season so that these data could be compared to fish and complexity data. Standard deviation of sediment depth (cm) was calculated to indicate how permanent (low standard deviation) or ephemeral (high standard deviation) sediment cover was on reefs.

### Statistical analyses

Statistical analyses were conducted in R version 3.2.0 [60]. We examined environmental variables for collinearity, and variables that were not collinear were retained for analyses. For example, water temperature and reef depth had a low correlation coefficient (0.04), so both were retained for subsequent analyses.

We used generalized linear models (GLMs) to determine the relationships between fish community metrics (abundance, biomass, and richness) and environmental variables and to specifically investigate how reef complexity influenced reef fishes. For fish abundance, we conducted GLMs with a negative-binomial error distribution and a log-link function using the ‘MASS’ package [64]. Fish abundance values from each reef were initially integers. Because we conducted two transects per reef during each sampling season, however, we later averaged the abundances from replicate transects to avoid pseudoreplication. Averaging resulted in non-integer abundances, so prior to performing GLMs, we rounded the mean abundance data to the nearest integer since we did not encounter fractions of fish and since the negative-binomial distribution requires integers. For fish biomass data, which are inherently continuous, we utilized a gamma distribution with a log link. For species richness data, which are integers, we used a Poisson distribution.

For each response variable (e.g., abundance, biomass, richness), we fit the most complex GLM first and then compared the most complex model to candidate models of reduced complexity until reaching the most parsimonious model. The most complex models regressed fish community metrics against a linear term (DRR) and squared term (DRR^2^) for complexity, as per methods in Allouche et al. [26] to determine whether fish community metrics and complexity exhibited a unimodal relationship. These complex models also included two environmental variables, depth and water temperature, to determine whether these additional abiotic factors helped explain variance in fish community metrics. We included an additional environmental variable, sediment standard deviation, exclusively for natural reefs.

Model selection from among our most complex and more parsimonious candidate models was conducted using Akaike information criterion (AIC) values based on minimum AIC. We conducted graphical and analytical assessments of fit to compare the predicted values from the model to the observed values. For graphical assessments of fit, we plotted the estimated probability distribution with the observed fish community metric values superimposed. This graphical method allowed us to determine whether the observed values appear typical of the estimated distribution. For analytical assessments of fit, we calculated *P*-values where the observed value of fish community metrics was treated as the test statistic and the predicted probability distribution was treated as the null model.

The magnitude of the coefficients for predictor variables and the associated *P*-values for the best model, as determined by AIC comparisons and both graphical and analytical assessments of fit, determined the type of relationship between fish community metrics and DRR: linear, quadratic, unimodal, or no relationship. If only the linear term (DRR) was significant, then a linear model was assumed. If only the quadratic term (DRR^2^) was significant, then a quadratic relationship was assumed. If both linear (DRR) and quadratic (DRR^2^) model terms were significant, then the relationship was categorized as unimodal [26]. If no term was significant, then this indicated no effect of DRR on fish community metrics. Models were evaluated separately by reef type: natural reefs and artificial reefs for total fishes and for each individual size class of fishes; small (1–10 cm), medium (11–29 cm), large (30–49 cm), extra large (*≥* 50 cm).

To evaluate whether fish community metrics varied by category of reef morphology, we used rugosity and *in situ* observations to classify natural reefs as either pavements-and-rubble or extensive ledges and artificial reefs as either low-relief concrete structures or complex ships. We calculated average fish abundance, biomass, and species richness for these four reef morphologies for all fishes. We tested for differences in fish community metrics by reef morphology using analysis of variance (ANOVA) followed by post-hoc pairwise t-tests. Abundance and biomass data were both log transformed to meet homogeneity of variance assumptions.

To determine whether fish community composition varied by reef morphology, we used permutational analysis of variance (PERMANOVA), nonmetric multidimensional scaling (nMDS) analysis, and indicator species analysis. These tests were applied to the square-root transformed fish abundance matrix at the family taxonomic level. PERMANOVA, a permutation-based technique that uses variance partitioning [65], explicitly tested whether fish community composition differed by morphological categories. PERMANOVA used Bray-Curtis distances and 1,000 permutations and accounted for reef morphology (pavement-and-rubble, ledge, concrete, ships) and was run using the ‘vegan’ package [66]. nMDS, an ordination method that summarizes patterns in the structure of multivariate datasets [62,67,68], was performed on the fish community data using the ‘vegan’ package [66]. Samples were mapped into ordination space using the ecological distances between samples ordered by rank terms. Bray-Curtis distances summarized pairwise distances among samples and helped overcome the problem of joint absences in species data [66]. A Shepard diagram ensured linearity between the ordination distance and Bray-Curtis distance. Biplots with samples colored by reef morphology and superimposed ellipses indicating 50% confidence intervals allowed visualization of the relationships among samples in ordination space. Indicator species analysis determined which species were indicators of the four classes of reef morphology and was performed with the ‘indicspecies’ package [69]. Weighted averages of the indicator families were projected on top of the sample space on the nMDS biplot to visualize community patterns by reef morphology.

## Results

We sampled a total of 246 transects on 30 temperate reefs located on the continental shelf of NC. Across the transects, 336,774 individual fishes belonging to 141 species and 47 families were observed (S2 Table). Total biomass of fishes was 43,570 kg. When two transects were conducted at a reef in a single season, the transects were averaged as a single replicate; results reported below correspond to these average values.

**Fig 2:**
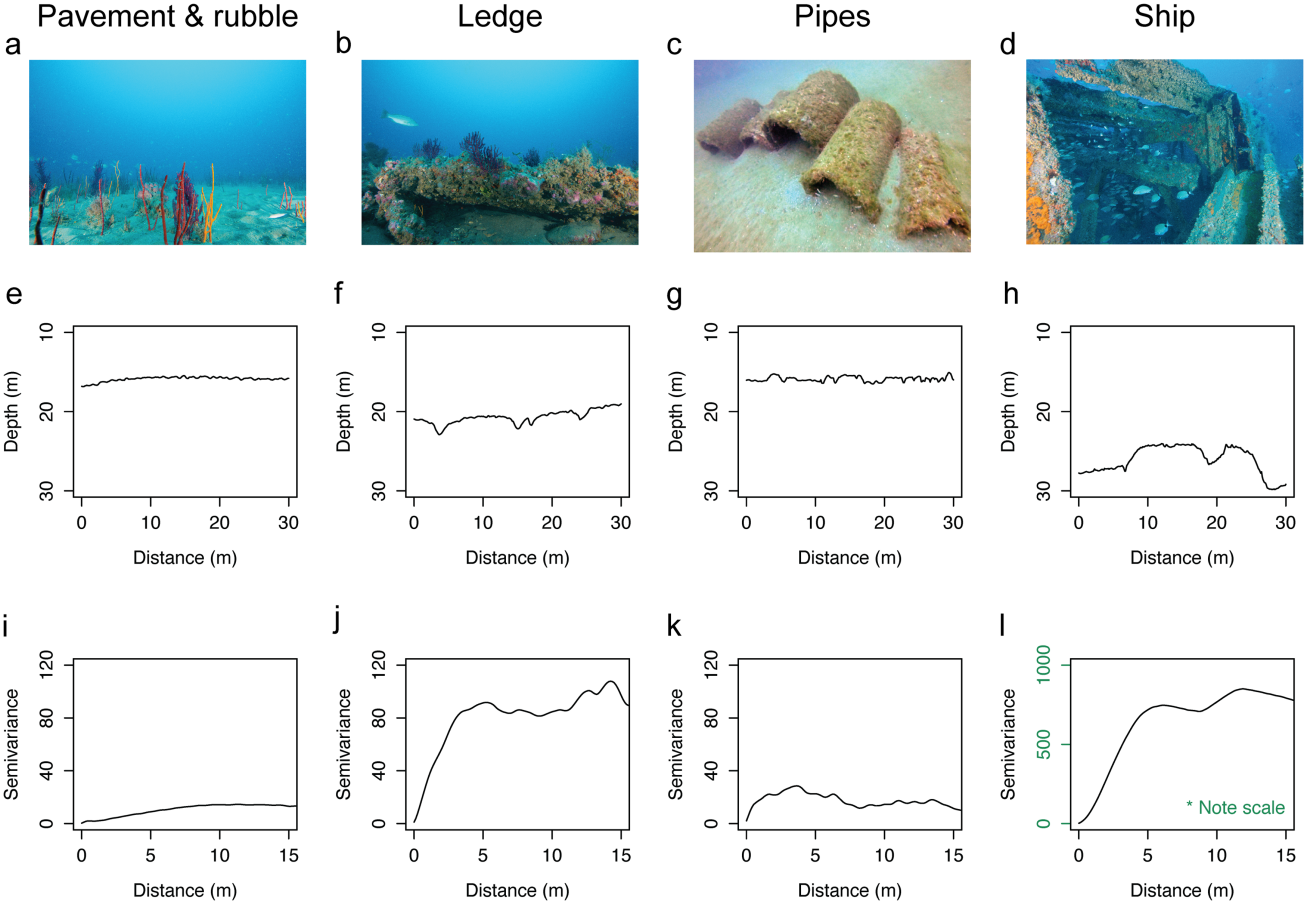
Habitat complexity of temperate reefs. a-d) Representative images of temperate reef morphologies. e-h) Representative depth contours of each reef morphology along the surveyed transect length. i-l) Representative semivariograms of each reef for half the distance of the surveyed transect length. Columns refer to different reef morphologies as follows, from left to right: naturally occurring pavement-and-rubble reef, naturally occurring ledge outcrop, artificial reef composed of concrete pipes, and a ship representative of historic shipwrecks and vessels intentionally sunk to enhance fish habitat.

Sampled reefs included both naturally occurring rocky reefs and human-made structures that varied in habitat complexity (Fig 2). Natural reefs ranged from flat pavements to distinct ledges (Fig 2a and 2b). Flat pavement-and-rubble reefs displayed relatively uniform contours (Fig 2e), with low variability in reef structure over the length of the transect along which fishes were surveyed (Fig 2i). Ledges, in contrast, contained either sharp or gradual drops and rises in reef height and exhibited higher spatial variability compared to the pavement-type reefs (Fig 2b, 2f and 2j). Artificial structures represented architecturally diverse habitats ranging from concrete pipes to shipwrecks and purposely scuttled vessels (Fig 2c and 2d). Structures nearly flush with the natural sandy seafloor, such as concrete pipes, displayed a relatively uniform contour map, where slight peaks in elevation coincided with the occurrence of human-made reef materials (Fig 2g), as well as low variability in structural complexity over transects (Fig 2k). Shipwrecks and purposely sunk vessels protruded into the water column forming pronounced peaks and valleys in their contours, characterized by greater variability than lower relief structures, such as concrete pipes (Fig 2h and 2l).

**Fig 3:**
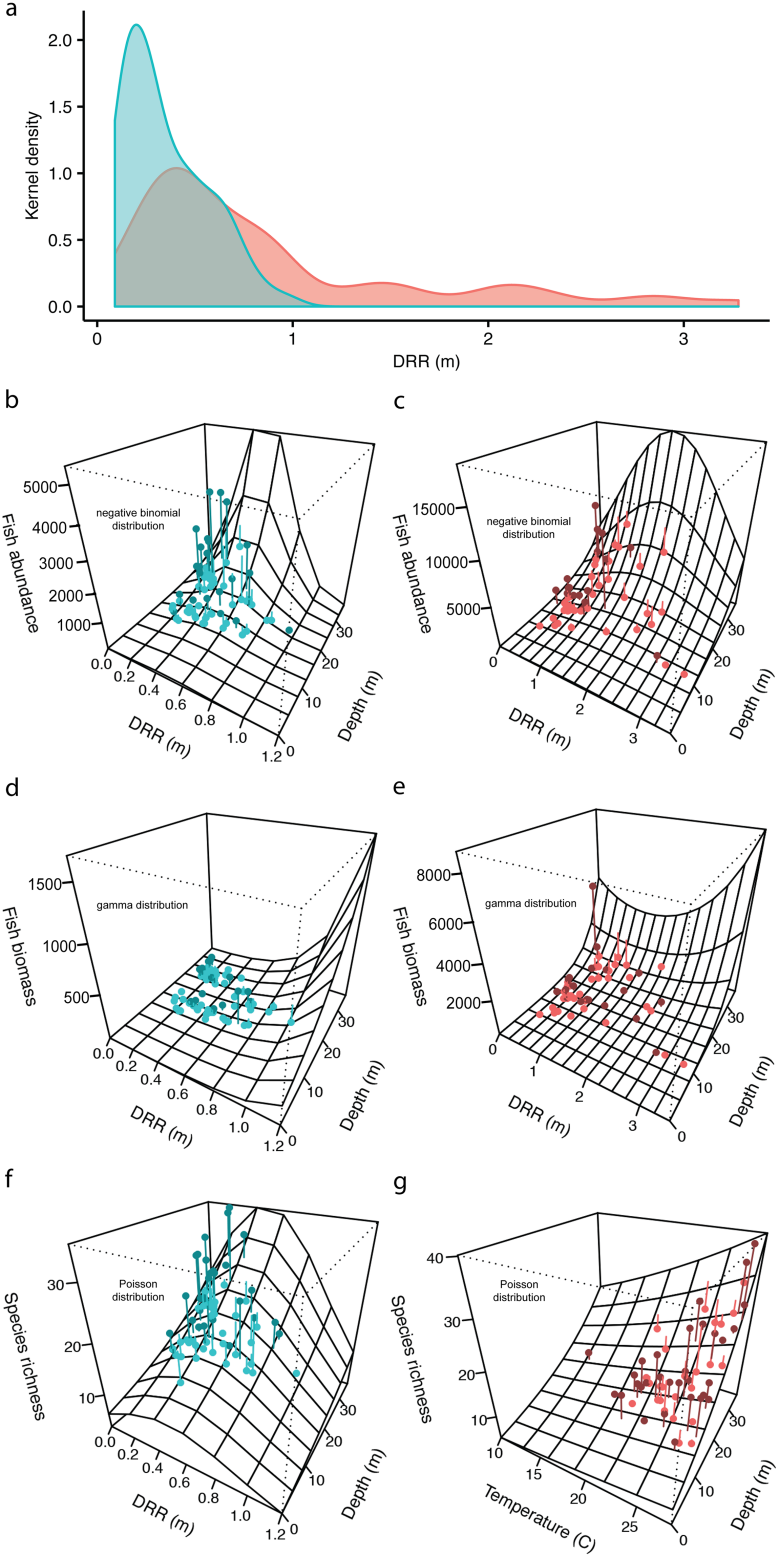
Relationship between digital reef rugosity (DRR) and fish community metrics on natural (blue) and artificial (red) temperate reefs. A) Kernel density of digital reef rugosity (DRR) by reef type (N_*natural*_ = 67, N_*artificial*_ = 56). B-G) Three-dimensional surface plot of GLM between fish community metrics and environmental predictor variables for natural reefs (left column) and artificial reefs (right column). Perspective grid surface represents GLM predictions. Points are raw data. Perpendicular segments attached to points depict whether the raw data are above (positive, dark color) or below (negative, light color) the surface predicted by GLM. Abundance (fishes / 120 m^*2*^) was modeled with a negative-binomial error distribution (b-c), biomass (kg / 120 m^*2*^) with a gamma distribution (d-e), and species richness with a Poisson distribution (f-g).

Complexity of both natural and artificial reefs was calculated with a DRR metric, such that low rugosity reflects low structural complexity and high rugosity coincides with high structural complexity. The distribution of rugosity for all reefs ranged from flat (0.1 m DRR minimum) to highly rugose (3.3 m DRR maximum; Fig 3a). The distribution of natural reefs centered on flatter rugosity values (0.1–1.0 m DRR) than those of artificial, which had a wider range (0.2–3.3 m DRR) weighted towards the more complex part of the rugosity spectrum. Temperate reefs on the continental shelf encompassed a wide variety of shapes and sizes but natural reefs occurred over the lower third of the range in complexity exhibited by artificial, human-made structures. Likewise, vertical relief, which was highly correlated with DRR (correlation 0.98), was greater for artificial reefs (1.0–8.7 m vertical relief) than for natural reefs (0.5–3.6 m vertical relief).

Intermediate levels of reef complexity maximized fish abundance for both natural and artificial reefs (Fig 3b and 3c). As complexity increased, fish abundance increased until reaching an inflection point at intermediate levels of reef complexity; when reef complexity surpassed intermediate levels (inflection point), fish abundance decreased. For naturally occurring reefs, in the GLM with a negative-binomial error distribution containing linear and quadratic terms for natural reef DRR and a linear term accounting for reef depths, all terms were significant (Table 1; DRR *P* < 0.0001; DRR^2^ *P* < 0.001; depth *P* < 0.0001), and the inflection point was within the range of the data, suggesting a unimodal curve (Fig 3b). The relationship between habitat complexity and fish abundance on artificial reefs was marginally unimodal and was most significantly influenced by reef depth (Table 1; DRR *P* < 0.04; DRR^2^ *P* < 0.08; depth *P* > 0.0001; Fig 3c). For biomass, neither the linear nor quadratic terms for DRR described the relationship with complexity across reef types (Table 1; biomass: DRR *P >* 0.05; DRR^2^ *P* > 0.05; Fig 3d and 3e). The model that contained DRR and DRR^2^, however, fit better than models excluding DRR terms, indicating that DRR did explain a small amount of variation in fish biomass. Regardless, reef depth explained the greatest amount of variation in fish biomass on both natural (Table 1; depth for natural reefs *P* = 0.05) and artificial reefs (Table 1; depth for artificial reefs *P* < 0.0001). On natural reefs, species richness displayed a unimodal relationship with reef complexity, when accounting for reef depth (Table 1; DRR *P* < 0.01; DRR^2^ *P* < 0.01; depth *P* < 0.0001; Fig 3f), whereas species richness was unrelated to DRR on artificial reefs where reef depth and water temperature positively influenced richness (Table 1; depth: *P* < 0.001; temperature: *P* < 0.01; Fig 3g).

**Table 1:** GLM results for the relationship between fish community metrics (abundance, biomass, richness) and environmental predictor variables by reef type. Environmental variables include digital reef rugosity (DRR (m)), squared digital reef rugosity (DRR ^*2*^ (m)), average reef depth (m), average water temperature (°C), and standard deviation of sediment cover (m) approximating sediment dynamics. Coefficients, standard error (SE), Z-values and *P*-values are provided for each environmental parameter. Bold values indicate significance or marginal significance. Interpretation of the pattern (unimodal or non-significant (NS)) between rugosity and the fish community metric are displayed for each model. Model results displayed here were from the best models that we evaluated.

Response variable	Reef type	Error distribution	Predictor variable	Coefficient	SE	Z-value	*P*-value	Rugosity pattern
Abundance	Natural	Negative binomial	Intercept	2.26	0.79	2.84	<0.01	Unimodal
			**DRR**	9.72	2.38	4.08	**<0.0001**	
			**DRR**^**2**^	-9.81	2.58	-3.81	**<0.001**	
			**Depth**	0.11	0.02	4.76	**<0.0001**	
Abundance	Artificial	Negative binomial	Intercept	4.37	0.45	9.72	<0.0001	Unimodal (marginally)
			**DRR**	1.31	0.63	2.07	**0.04**	
			**DRR**^**2**^	-0.37	0.21	-1.75	**0.08**	
			**Depth**	0.12	0.02	5.82	**<0.0001**	
Biomass	Natural	Gamma	Intercept	2.38	0.95	2.50	0.02	NS
			DRR	-0.17	2.86	-0.06	0.95	
			DRR^2^	2.24	3.10	0.72	0.47	
			**Depth**	0.06	0.02	2.00	**0.05**	
Biomass	Artificial	Gamma	Intercept	1.89	0.57	3.34	<0.01	NS
			DRR	-0.55	0.80	-0.68	0.49	
			DRR^2^	0.20	0.27	0.77	0.44	
			**Depth**	0.18	0.02	7.12	**<0.0001**	
Species richness	Natural	Poisson	Intercept	1.77	0.20	8.79	<0.0001	Unimodal
			**DRR**	2.22	0.60	3.69	**<0.001**	
			**DRR**^**2**^	-2.33	0.67	-3.49	**<0.001**	
			**Depth**	0.04	0.01	6.16	**<0.0001**	
Species richness	Artificial	Poisson	Intercept	1.28	0.28	4.54	<0.0001	NS
			**Depth**	0.03	0.01	6.37	**<0.0001**	
			**Temperature**	0.04	0.01	3.78	**<0.001**	

The relationship between fish abundance and reef structure differed by fish size class for each type (natural versus artificial) of temperate reef (S1 Fig; S3 Table). The unimodal relationship between complexity and abundance for natural reefs that occurred for total fishes (Fig 3) was replicated for just small fishes (1–10 cm) and also influenced by reef depth, water temperature, and sediment dynamics (S1a Fig), whereas abundances of just medium (11–29 cm) and just large fishes (30–49 cm) were unrelated to complexity but were related to depth and sediment, respectively (S1c Fig). A marginally significant linear, positive relationship described the abundance of extra-large (*≥* 50 cm) fishes as a function of complexity (S1d Fig; DRR *P* = 0.06), when accounting for reef depth where deeper reefs supported more extra-large fishes. For artificial reefs, the pattern of fish abundance having a unimodal relationship with habitat complexity for total fishes (Fig 3e) was preserved for just extra-large ((*≥* 50 cm) fishes, yet the inflection point occurred at lower measures of reef complexity for this size class of fishes compared to the curve for total fishes (S1h Fig). Abundance of large (30–49 cm) fishes was marginally and linearly related to complexity, when accounting for reef depth (S1g Fig). Small fish abundance was greater on deeper reefs, and medium fish abundance was greater on deeper and warmer temperature reefs, but abundance of both of these size classes was unrelated to complexity (S1e and S1f Fig).

**Fig 4:**
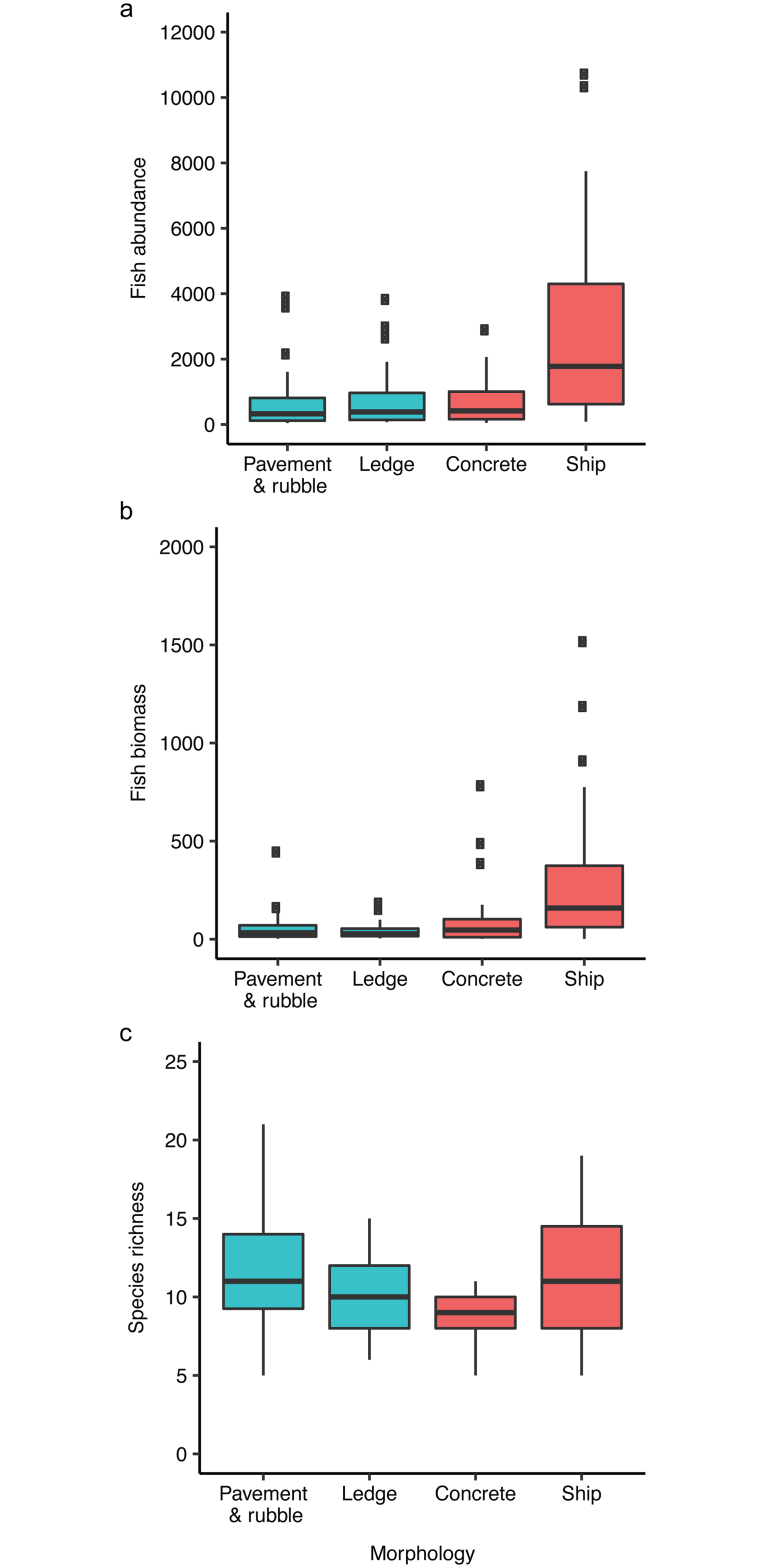
Fish community metrics by morphological category for natural reefs (blue; N_pavement&rubble_ = 38, N_ledge_ = 29) and artificial reefs (red; N_concrete_ = 17, N_ship_ = 39). A) Fish abundance (fishes per 120 m^2^). B) Fish biomass (kg / 120 m^2^). C) Fish species richness. Data displayed are untransformed, whereas ANOVAs were conducted on log-transformed data for abundance and biomass to meet assumptions of homogeneity of variance.

Because the unimodal relationship indicated that the least complex and most complex of each reef type were similar in numbers of fishes across a range of complexity values, we categorically compared the least versus most complex reefs by morphologies: pavement-and-rubble (natural); ledge (natural); concrete (artificial); ships (artificial). Abundance and biomass of fishes using flat and highly complex natural habitats and low complexity concrete habitats did not differ from each other but were substantially less than the number and biomass of fishes using ships for habitat (Fig 4a and 4b; ANOVA: abundance F_3,119_ = 11.13, *P* < 0.0001; biomass F_3,119_ = 9.12, *P* < 0.0001). Richness differed by reef morphology (Fig 4c; ANOVA, F_3,119_ = 4.33, *P* = 0.006). Flat and complex natural reefs supported equivalent numbers of species; however, complex artificial reefs (ships) supported more species than low complexity artificial reefs (concrete). Pavement-and-rubble reefs hosted higher species richness than concrete reefs.

**Fig 5:**
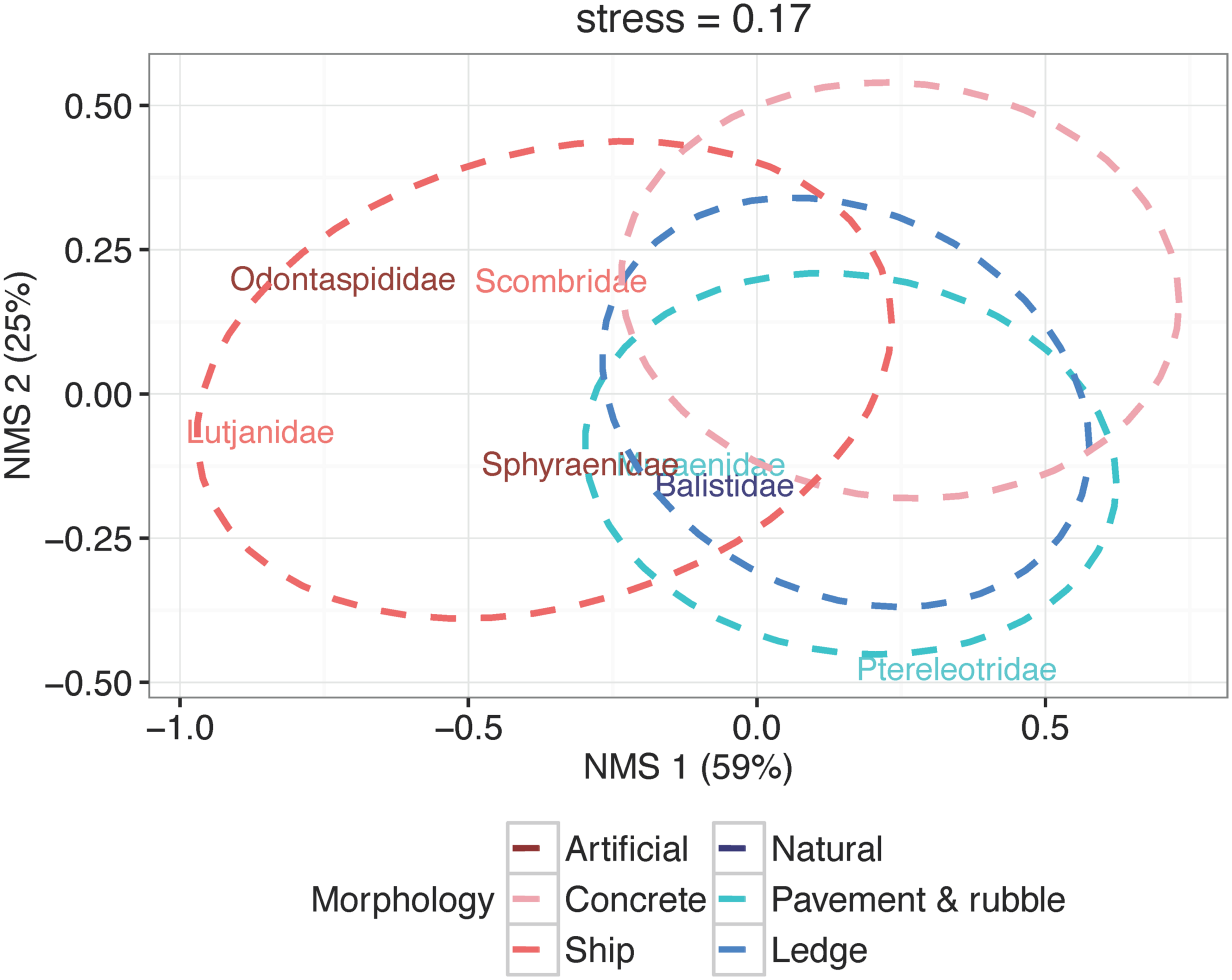
Biplot of nonmetric multidimensional scaling (nMDS) ordination for fish community at the family level overlaid with indicators of reef morphologies. Ellipses are 50% confidence intervals for samples classified by each reef morphology. Family names correspond to weighted averages of indicator families, colored according to morphology or reef type (artificial or natural).

Community compositions of fishes on pavement and ledge morphologies were similar (Fig 5), while the communities of fishes on low-lying concrete structures diverged from those of structurally unique ships (Fig 5; PERMANOVA: F_3,122_ = 4.00, *P* < 0.001). Balistidae (triggerfish; indicator value = 0.49; *P* = 0.018) occurred on both pavements and ledges, whereas Muraenidae (eels; indicator value = 0.42; *P* = 0.023) and Ptereleotidae (blue dartfish; indicator value = 0.43; *P* = 0.007) indicated pavements. There were no indicators exclusive of ledges. Diodontidae (porcupinefish; indicator value = 0.33; *P* = 0.043) characterized concrete artificial reefs, whereas pelagic Scombridae (mackerel; indicator value = 0.41; *P* = 0.023) and Lutjanidae (snapper; indicator value = 0.61; *P* = 0.001) signified submerged vessels. Sphyraenidae (barracuda; indicator value = 0.56; *P* = 0.015), Odontaspididae (sandtiger; indicator value = 0.45; *P* = 0.002), and Dasyatidae (whiptail stingray; indicator value = 0.30; *P* = 0.045) denoted artificial reefs regardless of topography.

## Discussion

We provide evidence that intermediate levels of warm-temperate reef complexity maximized fish abundance on natural and artificial reefs, as well as species richness on natural reefs, challenging the current paradigm that reefs of highest complexity support the most fishes and the most species of fishes. For naturally occurring rocky reefs, we discovered that flat pavement-and-rubble fields supported similar abundance, biomass, species richness, and community composition of fishes as pronounced ledges. Although low- and high- complexity artificial reefs supported equivalent numbers of fishes, artificial reefs composed of low-lying concrete structures hosted lower abundance, biomass, and species richness, as well as different community composition, than submerged metal vessels protruding high into the water-column. Our results suggest that habitat-focused management efforts should include reefs representative of a wide-variety of structural complexities, including both the most topographically complex reefs and those that are low-lying and often ephemeral EFH on the continental shelf.

Our finding that intermediate levels of reef complexity, as measured by reef rugosity, maximized fish abundance of warm-temperate reefs disagrees with the notion that the most complex reefs support the most abundant communities of fishes. We suggest several explanations for why we found a unimodal shape for the curve portraying the relationship between structural complexity and fish abundance. First, our study focused on a fine spatial scale (30 m transects). Many studies use broader spatial scales to examine landscape- or ecosystem- scale species-habitat patterns and find that broad-scale topographic complexity positively correlates with species abundance [9,70]. On temperate reefs on the continental shelf similar to those we evaluated, for example, fish abundance increases with increasing complexity values derived from multibeam bathymetry [53,71]. However, how fishes use their habitat changes across spatial scale [72]. Here, perhaps our choice in spatial scale illuminated a novel relationship between fish abundance and rugosity at a local spatial scale. Second, our study decoupled substrate type for natural reefs from an inherent productivity gradient. In most studies of habitat heterogeneity, substrate is not held constant, meaning that substrate type is coupled with an intrinsic productivity gradient [26]. In a terrestrial heterogeneity gradient, for example, substrate type could hypothetically stem from two distinct habitat types, deserts and high grass prairies, each with different substrates. Prairies have higher densities of plants by nature of their substrate than deserts, forming an inherent productivity gradient. In our study, however, because the substrate type remained consistent—rock substrate—for natural reefs, the raw substrate (excluding benthic community) for each reef type was decoupled from a respective productivity gradient. This decoupling allowed us to examine structural complexity independently from the habitat type, perhaps resulting in a unimodal curve for natural reefs for both abundance and species richness, whereas other studies found a positive relationship when coupling productivity and substrate type. For artificial reefs, however, the substrate type varied from concrete pipes to metal ships, perhaps explaining why we found a unimodal relationship for only one fish response metric, abundance. Third, we hypothesize that fishing pressure contributed to these relationships. Fishing pressure applied to complex reefs likely exceeds pressure on flat reefs because complex reefs create striking, permanent features on the seafloor that are easy for fishers to repeatedly locate on their bottom-finders, especially when using GPS units. In contrast, flat reefs, which are covered and exposed by sediment over time, form transient features difficult to locate with bottom-finders. Fishers can readily return to the same complex reefs, potentially reducing numbers of fishes and/or numbers of targeted species, such as apex predators. Reducing numbers of fishes and/or targeted species could drive decreased fish abundance on complex reefs. However, since we did not measure fishing pressure on the reefs, we were unable to quantitatively test the hypothesis that fishing pressure may shape the relationship between structural complexity and fish by decreasing fish abundance on the most complex reefs.

Fishes of different size classes responded differently to structural complexity. On natural reefs, abundance of total fishes, as well as just small fishes, had a unimodal relationship with complexity. Medium and large fishes were unrelated to structural complexity. Numbers of extra-large fishes, often including apex predators, such as *Mycteroperca microlepsis* (gag), *Mycteroperca phenax* (scamp), and *Seriola dumerili* (greater amberjack), increased linearly with rugosity on natural reefs, albeit with marginal statistical significance, concurring with previous temperate reef research [53,73,74]. On artificial reefs, total fishes and extra-large fishes only both exhibited a unimodal relationship with habitat complexity, while large fish were linearly related to complexity, and other size classes were unrelated. These size-class specific responses may be explained by inherent associations of fishes with habitat that change through ontogeny. For example, fish, such as *M*. *microlepsis*, move from shallow, nearshore habitats of typically lower complexity to offshore, deeper habitats of generally higher complexity as they mature [75,76]. Our data support this notion, as abundance of extra-large fishes on both natural and artificial reef types increased with reef depth. This change in habitat preference through ontogeny may explain differences in how fishes respond to complexity by size class.

The strength of the unimodal relationship between habitat complexity and fish abundance was stronger for natural than artificial reefs, and intermediate complexity maximized fish species richness on natural but not artificial reefs; we pose three explanations. First, artificial reefs harbored three-to-four times as many fishes as natural reefs, represented most prominently by schooling fishes. Schooling fishes, such as *Haemulon aurolineatum* (tomtate), *Rhomboplites aurorubens* (vermillion snapper), and *Decapterus* sp. (scad), drove the pattern of elevated fish abundance on artificial versus natural reefs. This supports previous findings that schooling fishes, including those that are partially planktivorous (*H*. *aurolineatum* and *R*. *aurorubens*; e.g., consume plankton and other prey items) and those that are strictly planktivores (*Decapterus* sp.), are more abundant on artificial than natural reefs [77,78]. Because presence of schooling fishes on reefs is more ephemeral than the presence of demersal fishes, variability introduced by schooling fishes may have allowed the expression of a less pronounced unimodal shape to the abundance-complexity curve on artificial as compared to natural reefs. Second, natural reef complexity clustered in the lower third of the value range of artificial reefs. Lower complexity of natural temperate reefs makes them susceptible to burial and exposure by sediment movement [46]. Low-lying artificial reefs, such as concrete pipes, face sediment movement similar to those experienced by natural reefs, however, vertically-extensive artificial reefs, such as metal ships, do not experience the same levels of sediment burial and exposure as their lower-relief counterparts. This discrepancy where low- and high- complexity artificial reefs face differing levels of physical disturbance could explain the weaker species-complexity relationship on artificial versus natural reefs. Third, although we surveyed an identical area on each reef, artificial reefs often occupy a smaller benthic footprint than natural reefs. Natural reefs often form extensive, branching networks, whereas artificial reefs act as discrete islands. The island-like nature of artificial reefs where habitat occupies discrete patches may contribute to a less pronounced species-complexity relationship for artificial reefs than for their natural reef counterparts. Assessing the relationship of fish community metrics and natural reef rugosity in the context of the arrangement of habitats on a larger scale represents a compelling avenue for future research.

Our finding that intermediate levels of complexity on natural reefs maximized fish species richness provides evidence that the area-heterogeneity tradeoff [25,26] operates on warm-temperate reefs. Ours is the first study, to our knowledge, suggesting that complexity of rocky, warm-temperate reefs reaches a threshold, above which species richness decreases. For abundance, the area-heterogeneity tradeoff predicts a negative relationship with increasing heterogeneity, depending on the system [26]. In our temperate system, we found a unimodal relationship between fish abundance and heterogeneity. We posit two explanations for why our results for abundance differ from theoretical expectations. First, the area-heterogeneity tradeoff is typically envisioned in a landcover-diversity context with heterogeneity indicating different habitat types. In our study, we measured heterogeneity of different reef types as DRR, a relevant metric for fishes across multiple scales [8,70], yet rugosity may not be as relevant at a landscape scale for the tradeoff hypothesis. Second, besides tradeoffs between the number of fishes and fish species that can be supported by different levels of habitat complexity, other tradeoffs likely occur on temperate reefs that are not area related. For example, we found that environmental variables, such as reef depth and water temperature, influenced fish communities, agreeing with previous temperate-reef research on the southeast US Atlantic continental shelf [79].

Naturally occurring pavement-and-rubble reefs harbored similar communities (abundance, biomass, richness, community composition) of fishes as did rocky ledges. Similar community types across natural reef morphologies is particularly interesting from a management perspective. This is because flat, pavement morphologies, often covered with a veneer of sediment, although federally designated as EFH prove difficult to detect [80], as they are frequently buried by sediment. Commonly employed seafloor mapping methods include side-scan sonar and multibeam bathymetry, which use sound waves to ensonify the seafloor topography [81]. Side-scan sonar cannot adequately detect pavements covered in a veneer of sand unless mapping is conducted at fine resolution to detect invertebrates, such as soft coral, that create a texture distinguishable from sand ([82], fine resolution </ = 4 m^2^). Multibeam bathymetry data can detect pavements if researchers elect to use backscatter data [83,84]. One effective way to detect pavements couples typical habitat-mapping data with fisheries acoustics data [53,85,86]. Using this combined method, if larger than expected concentrations of fishes occur above an area seemingly devoid of rocky reefs, then researchers investigate whether these pavement-type reefs covered in a veneer of sediment may be responsible for the elevated numbers and/or biomass of fishes. Other instruments, such as video cameras and sub-bottom profilers, as well as *in situ* diver-based visual surveys, can easily detect pavements [83,87]. However, the sampling area of these instruments is so small that for surveys of large geographic areas (> 10–100’s of m), these methods prove inefficient. Novel methods to detect flat pavements should be developed given that these low-lying habitats support similar numbers and types of fishes as ledges.

Low- and high- complexity artificial reefs harbored similar numbers of fishes as a function of the continuous predictor DRR, but when artificial reefs were separated into morphological categories of low-lying concrete structures and metal ships, the pattern differed. Concrete pipes hosted fewer fish and species of fish, as well as distinct community types, compared to metal ships. Concrete structures nearly flush with the sandy seafloor formed prime habitat for demersal fishes, such as Diodontidae (porcupinefish), that prey on animals growing on reefs and living within the sediment and also use the reef structure to seek refuge from predators. Pelagic species often found in the water column above reefs, including Scombridae (mackerel) and Lutjanidae (snapper), however, preferred ships with vertically-extensive topography. Three families of top predators, Odontaspididae (sand-tiger sharks), Sphyraenidae (barracuda), and Dasyatidae (whiptail stingrays) indicated generic artificial reefs. Distinguishable communities of fishes relying on low- versus high-complexity artificial reefs suggest that managers should deploy human-made reefs of varying topographic complexity based on particular fisheries they aim to enhance. Renewable energy infrastructure, such as wind turbine monopiles that extend throughout the water column (high-complexity) and associated anti-scour aprons of rocks and concrete (low-complexity), may combine attributes from reefs across a range of complexities, providing habitat for both demersal and pelagic reef-associated communities. Additionally, given similarities in fish community composition between low-lying concrete pipes and natural reefs, concrete pipes may serve as refugia for fishes commonly occupying natural reefs in the future.

Marine conservation and management initiatives commonly target the most structurally complex and diverse reefs. Our results, however, suggest that less complex habitats require as much consideration for these initiatives as more complex morphologies. This is a pressing issue as human uses of the coastal ocean increase and marine-spatial planning becomes more commonplace along the continental shelf. Management efforts should afford equal consideration to a diversity of reef types, including both low- and high-complexity reefs. Given current difficulties in detecting naturally occurring rock pavements covered with a veneer of sediment, these flat reefs, even though already designated as EFH, warrant extra attention when obtaining data used for spatially-explicit planning during seafloor mapping so that they can be delineated. Submerged, human-made structures across a range of topographies support different communities of fishes, and this information will prove useful when designing and deploying additional unnatural structures.

## Supporting information

S1 Fig Response of fish abundance to digital reef rugosity (DRR) by reef type and fish size class. Color denotes reef type: natural reefs (blue; a-d) and artificial reefs (red; e-h). Row indicates fish size classes: a and e) small (1–10 cm) fishes; b and f) medium (11–29 cm) fishes; c and g) large (30–49 cm) fishes; d and h) extra-large fishes (*≥* 50 cm). Solid lines represent unimodal relationships between DRR and fish abundance (DRR: *P* < 0.05, DRR^2^: *P* < 0.05), whereas absence of a line indicates a non-significant relationship between DRR and fish abundance and a dashed line indicates a marginally-significant relationship.
(DOCX)

S1 Table Descriptions of thirty reefs surveyed. Mean environmental variables include digital reef rugosity (DRR), vertical relief (relief), depth, water temperature (temp), sediment standard deviation (sed; natural reefs only), and location. Date indicates month and year (month/year) of replicate transects.
(DOCX)

S2 Table Species list from 246 fish belt-transects conducted on warm-temperate reefs of the NC continental shelf. Bold text indicates fishes in the federally managed snapper-grouper complex. Abundance values indicate the total numbers of each species observed across the 246 transects, as well as the number of each species observed on natural and artificial reefs.
(DOCX)

S3 Table GLM results for the relationship between fish abundance and environmental predictor variables by reef type and fish size class. Environmental variables include digital reef rugosity (DRR (m)), squared digital reef rugosity (DRR ^2^ (m)), average reef depth (m), average water temperature (°C), and standard deviation of sediment cover (m) approximating sediment dynamics. Coefficients, standard error (SE), Z-values and *P*-values are provided for each environmental parameter. Bold values indicate significance. Interpretation of the pattern (unimodal, linear, or non-significant (NS)) between rugosity and the fish abundance are displayed for each model. Model results displayed here were from the best models that we evaluated.
(DOCX)

## References

[1] MacArthur RH, MacArthur JW. On bird species diversity. Ecology. 1961;42: 594–598. Available: http://www.esajournals.org/doi/abs/10.2307/1932254

[2] Kovalenko KE, Thomaz SM, Warfe DM. Habitat complexity: approaches and future directions. Hydrobiologia. 2012;685: 1–17. doi: 10.1007/s10750-011-0974-z

[3] Jung K, Kaiser S, Böhm S, Nieschulze J, Kalko EK V. Moving in three dimensions: effects of structural complexity on occurrence and activity of insectivorous bats in managed forest stands. J Appl Ecol. 2012;49: 523–531. doi: 10.1111/j.1365-2664.2012.02116.x

[4] Khanaposhtani MG, Kaboli M, Karami M, Etemad V. Effect of habitat complexity on richness, abundance and distributional pattern of forest birds. Environ Manage. 2012;50: 296–303. doi: 10.1007/s00267-012-9877-7 22661015 10.1007/s00267-012-9877-7

[5] Gorman OT, Karr JR. Habitat structure and stream fish communities. Ecology. 1978;59: 507–515. Available: http://www.jstor.org/stable/1936581

[6] Schneider KN, Winemiller KO. Structural complexity of woody debris patches influences fish and macroinvertebrate species richness in a temperate floodplain-river system. Hydrobiologia. 2008;610: 235–244. doi: 10.1007/s10750-008-9438-5

[7] McCormick MI. Comparison of field methods for measuring surface topography and their associations with a tropical reef fish assemblage. Mar Ecol Prog Ser. 1994;112: 87–96. Available: http://www.reeffishecology.com/files/file/McCormick94-topo.pdf

[8] Dustan P, Doherty O, Pardede S. Digital reef rugosity estimates coral reef habitat complexity. PLoS One. 2013;8: e57386 doi: 10.1371/journal.pone.0057386 23437380 10.1371/journal.pone.0057386 PMC3578865

[9] Tews J, Brose U, Grimm V, Tielbörger K, Wichmann MC, Schwager M, et al Animal species diversity driven by habitat heterogeneity/diversity: the importance of keystone structures. J Biogeogr. 2004;31: 79–92. doi: 10.1046/j.0305-0270.2003.00994.x

[10] Holt RD. Spatial heterogeneity, indirect interactions, and the coexistence of prey species. Am Nat. 1984;124: 377–406. 10.1086/284280 29519131

[11] Huffaker CB. Experimental studies on predation: dispersion factors and predator-prey oscillations. Hilgardia. 1958;27: 343–383.

[12] Connell SD, Jones GP. The influence of habitat complexity on postrecruitment processes in a temperate reef fish population. J Exp Mar Bio Ecol. 1991;151: 271–294. Available: http://www.sciencedirect.com/science/article/pii/002209819190129K

[13] Almany GR. Differential effects of habitat complexity, predators and competitors on abundance of juvenile and adult coral reef fishes. Oecologia. 2004;141: 105–13. doi: 10.1007/s00442-004-1617-0 15197644 10.1007/s00442-004-1617-0

[14] Gilinsky E. The role of fish predation and spatial heterogeneity in determining benthic community structure. Ecology. 1984;65: 455–468.

[15] Gotceitas V, Colgan P. Predator foraging success and habitat complexity: quantitative test of the threshold hypothesis. Oecologia. 1989;80: 158–166. Available: http://link.springer.com/article/10.1007/BF00380145 28313101 10.1007/BF00380145

[16] Beukers JS, Jones GP. Habitat complexity modifies the impact of piscivores on a coral reef fish population. Oecologia. 1997;114: 50–59. Available: http://link.springer.com/article/10.1007/s004420050419 10.1007/s004420050419 28307557

[17] Crowder LB, Cooper WE. Habitat structural complexity and the interaction between bluegills and their prey. Ecology. 1982;63: 1802–1813. Available: http://www.jstor.org/stable/1940122

[18] Diehl S. Fish predation and benthic community structure: the role of omnivory and habitat complexity. Ecology. 1992;73: 1646–1661. Available: http://www.jstor.org/stable/1940017

[19] Grabowski JH. Habitat complexity distrupts predator-prey interactions but not the trohpic cascade on oyster reefs. Ecology. 2004;85: 995–1004. Available: http://www.esajournals.org/doi/abs/10.1890/03-0067

[20] Gazol A, Tamme R, Price JN, Hiiesalu I, Laanisto L, Pärtel M. A negative heterogeneity-diversity relationship found in experimental grassland communities. Oecologia. 2013;173: 545–555. doi: 10.1007/s00442-013-2623-x 23468237 10.1007/s00442-013-2623-x

[21] Hutchinson GE. Concluding remarks. Popul Stud Anim Ecol Demogr Cold Spring Harb Symp Quant Biol. 1957;22: 415–427.

[22] MacArthur RH, Wilson EO. An equilibrium theory of insular zoogeography. Evolution (N Y). 1963;17: 373–387.

[23] MacArthur RH. The theory of island biogeography. Volume 1 Princeton Univeristy Press; 1967.

[24] Simberloff DS, Wilson EO. Experimental zoogeography of islands. A two-year record of colonization. Ecology. 1970;51: 934–937.

[25] Kadmon R, Allouche O. Integrating the effects of area, isolation, and habitat heterogeneity on species diversity: a unification of island biogeography and niche theory. Am Nat. 2007;170: 443–454. doi: 10.1086/519853 17879194 10.1086/519853

[26] Allouche O, Kalyuzhny M, Moreno-Rueda G, Pizarro M, Kadmon R. Area-heterogeneity tradeoff and the diversity of ecological communities. Proc Natl Acad Sci. 2012;109: 17495–17500. doi: 10.1073/pnas.1208652109 23045670 10.1073/pnas.1208652109 PMC3491518

[27] Hortal J, Carrascal LM, Triantis KA, Thébault E, Meiri S, Sfenthourakis S. Species richness can decrease with altitude but not with habitat diversity. Proc Natl Acad Sci U S A. 2013;110: E2149–E2150. doi: 10.1073/pnas.1301663110 23661060 10.1073/pnas.1301663110 PMC3683747

[28] Carnicer J, Brotons L, Herrando S, Sol D. Improved empirical tests of area-heterogeneity tradeoffs. Proc Natl Acad Sci U S A. 2013;110: E2858–E2860. doi: 10.1073/pnas.1222681110 23754368 10.1073/pnas.1222681110 PMC3732947

[29] Seiferling I, Proulx R, Wirth C. Disentangling the environmental-heterogeneity—species-diversity relationship along a gradient of human footprint. Ecology. 2014;95: 2084–2095. doi: 10.1890/13-1344.1 25230461 10.1890/13-1344.1

[30] Pauly D, Christensen V, Dalsgaard J, Froese R, Torres FJ. Fishing down marine food webs. Science (80-). 1998;279: 860–863. doi: 10.1126/science.279.5352.860 10.1126/science.279.5352.860 9452385

[31] Jackson JBC, Kirby MX, Berger WH, Bjorndal KA, Botsford LW, Bourque BJ, et al Historical overfishing and the recent collapse of coastal ecosystems. Science (80-). 2001;293: 629–638. doi: 10.1126/science.1059199 11474098 10.1126/science.1059199

[32] Martínez ML, Intralawan A, Vázquez G, Pérez-Maqueo O, Sutton P, Landgrave R. The coasts of our world: ecological, economic and social importance. Ecol Econ. 2007;63: 254–272. doi: 10.1016/j.ecolecon.2006.10.022

[33] Arkema KK, Verutes GM, Wood SA, Clarke-Samuels C, Rosado S, Canto M, et al Embedding ecosystem services in coastal planning leads to better outcomes for people and nature. Proc Natl Acad Sci. 2015;112: 7390–7395. doi: 10.1073/pnas.1406483112 26082545 10.1073/pnas.1406483112 PMC4475972

[34] Cronk QCB. Islands: stability, diversity, conservation. Biodivers Conserv. 1997;6: 477–493. doi: 10.1023/A:1018372910025

[35] McCann KS. The diversity-stability debate. Nature. 2000;405: 228–233. doi: 10.1038/35012234 10821283 10.1038/35012234

[36] Lawler JJ, Aukema JE, Grant JB, Halpern BS, Kareiva P, Nelson CR, et al Conservation science: a 20-year report card. Front Ecol Environ. 2006;4: 473–480.

[37] Worm B, Barbier EB, Beaumont N, Duffy JE, Folke C, Halpern BS, et al Impacts of biodiversity loss on ocean ecosystem services. Science (80-). 2006;314: 787–790. doi: 10.1126/science.1132294 17082450 10.1126/science.1132294

[38] National Oceanic and Atmospheric Administration. Florida Keys National Marine Sanctuary final management plan / environmental impact statement. 1996;I: 1–809. http://sanctuaries.noaa.gov/library/pdfs/fknms_fmpfeis_1996.pdf

[39] Bohnsack JA. Consensus development and the use of marine reserves in the Florida Keys, U.S.A. Proc 8th Int Coral Reef Symp. 1997;2: 1927–1930.

[40] Riggs SR, Snyder SW, Hine AC, Mearns DL. Hardbottom morphology and relationship to the geologic framework: mid-Atlantic continental shelf. J Sediment Res. 1996;66: 830–846. Available: http://jsedres.sepmonline.org/content/66/4/830.short

[41] Riggs SR, Ambrose WG Jr., Cook JW, Snyder SW, Snyder SW. Sediment production on sediment-starved continental margins: the interrelationship between hardbottoms, sedimentological and benthic community processes, and storm dynamics. J Sediment Res. 1998;68: 155–168. Available: http://jsedres.sepmonline.org/content/68/1/155.short

[42] Stick D. Graveyard of the Atlantic: Shipwrecks of the North Carolina Coast. Univeristy of North Carolina Press; 1989.

[43] North Carolina Department of Natural Resources and Community Development—Division of Marine Fisheries. North Carolina Artificial Reef Master Plan. 1988.

[44] Gregg K, Murphey S. The role of vessels as artificial reef material on the Atlantic and Gulf of Mexico coasts of the United States. Special Report No. 38 of the Atlantic States Marine Fisheries Commission [Internet]. Morehead City, NC; 1994. http://www.asmfc.org/uploads/file/sr38RoleOfVesselsAsArtificialReefMaterial.pdf

[45] Renaud PE, Ambrose WG Jr., Riggs SR, Syster DA. Multi-level effects of severe storms on an offshore temperate reef system: benthic sediments, macroalgae, and implications for fisheries. Mar Ecol. 1996;17: 383–398. doi: 10.1111/j.1439-0485.1996.tb00516.x

[46] Renaud PE, Riggs SR, Ambrose WG Jr., Schmid K, Snyder SW. Biological-geological interactions: storm effects on macroalgal communities mediated by sediment characteristics and distribution. Cont Shelf Res. 1997;17: 37–56. Available: http://www.sciencedirect.com/science/article/pii/0278434396000192

[47] Renaud PE, Syster DA, Ambrose WG Jr.. Recruitment patterns of continental shelf benthos off North Carolina, USA: effects of sediment enrichment and impact on community structure. J Exp Mar Bio Ecol. 1999;237: 89–106. Available: http://www.sciencedirect.com/science/article/pii/S0022098198002226

[48] South Atlantic Fishery Management Council. Fishery management plan, regulatory impact review, and final environmental impact statement for the snapper-grouper fishery of the South Atlantic region [Internet]. Prepared by the South Atlantic Fishery Management Council in cooperation with National Marine Fisheries Service; 1983 pp. 1–303. http://safmc.net/wp-content/uploads/2016/06/SnapGroupFMP-1.pdf

[49] South Atlantic Fishery Management Council. Snapper Grouper Management Complex: Species Managed by the South Atlantic Fishery Management Council [Internet]. 2016 [cited 1 Jan 2016] pp. 1–3. http://safmc.net/wp-content/uploads/2016/06/SAFMC_SnapperGrouperManagedSpecies_091614.pdf

[50] Stephan CD, Lindquist DG. A comparative analysis of the fish assemblages associated with old and new shipwrecks and fish aggregating devices in Onslow Bay, North Carolina. Bull Mar Sci. 1989;44: 698–717. Available: http://www.ingentaconnect.com/content/umrsmas/bullmar/1989/00000044/00000002/art00014

[51] Parker RO Jr., Dixon RL. Changes in a North Carolina reef fish community after 15 years of intense fishing—global warming implications. Trans Am Fish Soc. 1998;127: 908–920.

[52] Deaton AS, Chappell WS, Hart K, O’Neal J, Boutin B. North Carolina Coastal Habitat Protection Plan. North Carolia Department of Environment and Natural Resources, Division of Marine Fisheries, Morehead City, NC; 2010.

[53] Taylor JC, Paxton AB, Voss CM, Sumners B, Buckel CA, Vander Pluym J, et al. Benthic habitat mapping and assessment in the Wilmington-East wind energy call area. OCS Study BOEM 2016–003 and NOAA Technical Memorandum 196. Atlantic OCS Region, Sterling, VA; 2016.

[54] Brock VE. A preliminary report on a method of estimating reef fish populations. J Wildl Manage. 1954;18: 297–308. Available: http://www.jstor.org/stable/3797016

[55] Brock RE. A critique of the visual census method for assessing coral reef fish populations. Bull Mar Sci. 1982;32: 269–276. Available: http://www.ingentaconnect.com/content/umrsmas/bullmar/1982/00000032/00000001/art00019

[56] Samoilys MA, Carlos G. Determining methods of underwater visual census for estimating the abundance of coral reef fishes. Environ Biol Fishes. 2000;57: 289–304. Available: http://link.springer.com/article/10.1023/A:1007679109359

[57] Froese R, Pauly D. FishBase [Internet]. 2016. www.fishbase.org

[58] Risk MJ. Fish diversity on a coral reef in the Virgin Islands. Atoll Res Bull. 1972; 1–4. Available: http://www.aoml.noaa.gov/general/lib/CREWS/Cleo/St.John/St_John2.pdf

[59] Sheather SJ, Jones MC. A reliable data-based bandwidth selection method for kernel density estimation. J R Stat Soc Ser B. 1991;53: 683–690.

[60] R Development Core Team. R: A language and environment for statistical computing [Internet]. R Foundation for Statistical Computing, Vienna, Austria; 2015 http://www.r-project.org/

[61] Legendre P, Fortin MJ. Spatial pattern and ecological analysis. Vegetatio. 1989;80: 107–138. doi: 10.1007/BF00048036

[62] Legendre P, Legendre L. Numerical ecology. 3rd Ed Amsterdam, the Netherlands: Elsevier; 2012.

[63] Isaaks EH, Srivastava RM. Applied Geostatistics. New York, New York, USA: Oxford University Press, Inc.; 1989.

[64] Venables MN, Ripley BD. Modern Applied Statistics with S. 4th Ed Springer, New York; 2002.

[65] Anderson MJ. A new method for non-parametric multivariate analysis of variance. Austral Ecol. 2001;26: 32–46. Available: http://onlinelibrary.wiley.com/doi/10.1111/j.1442-9993.2001.01070.pp.x/full

[66] Oksanen J, Guillaume Blanchet F, Kindt R, Legendre P, Minchin PR, O’Hara RB, et al. vegan: Community Ecology Package. 2015;R package. http://cran.r-project.org/package=vegan

[67] Shepard RN. The analysis of proximities: multidimensional scaling with an unknown distance function. I. Psychometrika. 1962;27: 125–140. Available: http://link.springer.com/article/10.1007/BF02289630

[68] Kruskal JB. Multidimensional scaling by optimizing goodness of fit to a nonmetric hypothesis. Psychometrika. 1964;29: 1–27. Available: http://link.springer.com/article/10.1007/BF02289565

[69] De Cáceres M, Legendre P. Associations between species and groups of sites: indices and statistical inference. Ecology. 2009;90: 3566–3574. doi: 10.1890/08-1823.1 20120823 10.1890/08-1823.1

[70] Dunn DC, Halpin PN. Rugosity-based regional modeling of hard-bottom habitat. Mar Ecol Prog Ser. 2009;377: 1–11. doi: 10.3354/meps07839

[71] Kracker L, Kendall M, McFall G. Benthic Features as a Determinant for Fish Biomass in Gray’s Reef National Marine Sanctuary. Mar Geod. 2008;31: 267–280. doi: 10.1080/01490410802466611

[72] Arias-González JE, Done TJ, Page CA, Cheal AJ, Kininmonth S, Garza-Pérez JR. Towards a reefscape ecology: relating biomass and trophic structure of fish assemblages to habitat at Davies Reef, Australia. Mar Ecol Prog Ser. 2006;320: 29–41. doi: 10.3354/meps320029

[73] Kracker L, Kendall M, McFall G. Benthic features as a determinant for fish biomass in Gray’s Reef National Marine Sanctuary. Mar Geod. 2008;31: 267–280. doi: 10.1080/01490410802466611

[74] Kendall MS, Bauer LJ, Jeffrey CFG. Influence of hard bottom morphology on fish assemblages of the continental shelf off Georgia, Southeastern USA. Bull Mar Sci. 2009;84: 265–286.

[75] Ross SW, Moser ML. Life history of juvenile gag, Mycteroperca microlepsis, in North Carolina estuaries. Bull Mar Sci. 1995;56: 222–237.

[76] Stallings CD, Coleman FC, Koenig CC, Markiewicz DA. Energy allocation in juveniles of a warm-temperate reef fish. Environ Biol Fishes. 2010;88: 389–398. doi: 10.1007/s10641-010-9655-4

[77] Arena PT, Jordan LKB, Spieler RE. Fish assemblages on sunken vessels and natural reefs in southeast Florida, USA. Hydrobiologia. 2007;580: 157–171. doi: 10.1007/s10750-006-0456-x

[78] Simon T, Joyeux J-C, Pinheiro HT. Fish assemblages on shipwrecks and natural rocky reefs strongly differ in trophic structure. Mar Environ Res. Elsevier Ltd; 2013;90: 55–65. doi: 10.1016/j.marenvres.2013.05.012 23796542 10.1016/j.marenvres.2013.05.012

[79] Whitfield PE, Muñoz RC, Buckel CA, Degan BP, Freshwater DW, Hare JA. Native fish community structure and Indo-Pacific lionfish *Pterois volitans* densities along a depth-temperature gradient in Onslow Bay, North Carolina, USA. Mar Ecol Prog Ser. 2014;509: 241–254. doi: 10.3354/meps10882

[80] Lucieer V, Hill NA, Barrett NS, Nichol S. Do marine substrates “look” and “sound” the same? Supervised classification of multibeam acoustic data using autonomous underwater vehicle images. Estuar Coast Shelf Sci. Elsevier Ltd; 2013;117: 94–106. doi: 10.1016/j.ecss.2012.11.001

[81] Simmonds J, MacLennan DN. Fisheries Acoustics: Theory and Practice. 2nd Ed Oxford, UK: Blackwell Science; 2005.

[82] Prada MC, Appeldoorn RS, Rivera JA. The effects of minimum map unit in coral reefs maps generated from high resolution side scan sonar mosaics. Coral Reefs. 2008;27: 297–310. doi: 10.1007/s00338-007-0328-5

[83] Kendall MS, Jensen OP, Alexander C, Field D, McFall G, Bohne R, et al Benthic mapping using sonar, video transects, and an innovative approach to accuracy assessment: a characterization of bottom features in the Georgia Bight. J Coast Res. 2005;21: 1154–1165. doi: 10.2112/03-0101R.1

[84] Kloser RJ, Penrose JD, Butler AJ. Multi-beam backscatter measurements used to infer seabed habitats. Cont Shelf Res. Elsevier; 2010;30: 1772–1782. doi: 10.1016/j.csr.2010.08.004

[85] Taylor JC, Ebert E. Mapping coral reef fish schools and aggregations with high-frequency multibeam and split-beam sonars. Acoust Soc Am Proc Meet Acoust. 2012; 1–9. doi: 10.1121/1.4772586

[86] Kracker LM, Taylor JC, Ebert EF, Battista TA, Menza C. Integration of fisheries acoustics surveys and bathymetric mapping to characterize midwater-seafloor habitats of US Virgin Islands and Puerto Rico (2008–2010). NOAA Technical Memorandum NOS NCCOS 130. 44pp. 2011.

[87] Walker BK, Riegl B, Dodge RE. Mapping coral reef habitats in southeast Florida using a combined technique approach. J Coast Res. 2008;24: 1138–1150. doi: 10.2112/06-0809.1

