# Supplementary material for: Flat and complex temperate reefs provide similar support for fish: Evidence for a unimodal species-habitat relationship
Source: PLoS One. 2017 Sep 5;12(9):e0183906. doi: 10.1371/journal.pone.0183906 (PMC5584758; doi:10.1371/journal.pone.0183906)
Supplement: S1 Fig — Color denotes reef type: natural reefs (blue; a-d) and artificial reefs (red; e-h). Row indicates fish size classes: a and e) small (1–10 cm) fishes; b and f) medium (11–29 cm) fishes; c and g) large (30–49 cm) fishes; d and h) extra-large fishes (≥ 50 cm). Solid lines represent unimodal relationships between DRR and fish abundance (DRR: P < 0.05, DRR2: P < 0.05), whereas absence of a line indicates a non-significant relationship between DRR and fish abundance and a dashed line indicates a marginally-significant relationship. (DOCX) [file pone.0183906.s001.docx]

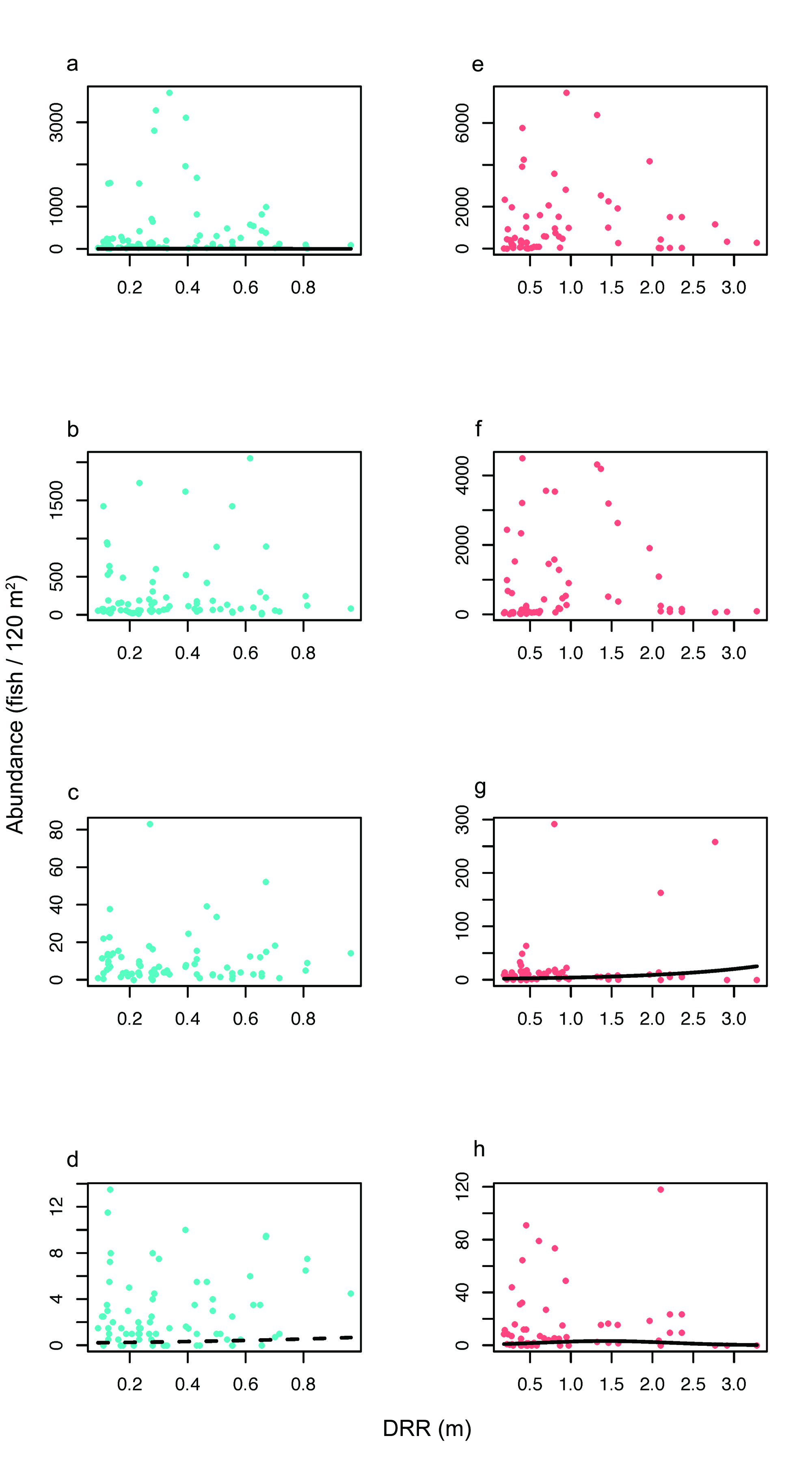


**S1 Fig: Response of fish abundance to digital reef rugosity (DRR) by reef type and fish size class. C**olor denotes reef type: natural reefs (blue; a-d) and artificial reefs (red; e-h). Row indicates fish size classes: a and e) small (1-10 cm) fishes; b and f) medium (11-29 cm) fishes; c and g) large (30-49 cm) fishes; d and h) extra-large fishes (*≥* 50 cm). Solid lines represent unimodal relationships between DRR and fish abundance (DRR: *P* < 0.05, DRR^2^: *P* < 0.05), whereas absence of a line indicates a non-significant relationship between DRR and fish abundance and a dashed line indicates a marginally-significant relationship.
