# Supplementary material for: Flat and complex temperate reefs provide similar support for fish: Evidence for a unimodal species-habitat relationship
Source: PLoS One. 2017 Sep 5;12(9):e0183906. doi: 10.1371/journal.pone.0183906 (PMC5584758; doi:10.1371/journal.pone.0183906)
Supplement: S1 Table — Mean environmental variables include digital reef rugosity (DRR), vertical relief (relief), depth, water temperature (temp), sediment standard deviation (sed; natural reefs only), and location. Date indicates month and year (month/year) of replicate transects. (DOCX) [file pone.0183906.s002.docx]

**S1 Table: Descriptions of thirty reefs surveyed.** Mean environmental variables include digital reef rugosity (DRR), vertical relief (relief), depth, water temperature (temp), sediment standard deviation (sed; natural reefs only), and location. Date indicates month and year (month/year) of replicate transects.

| **reef_name** | **reef_type** | **morphology** | **DRR (m)** | **relief (m)** | **depth (m)** | **temp (^o^C)** | **sed**  **(cm)** | **location** | **date** |
| --- | --- | --- | --- | --- | --- | --- | --- | --- | --- |
| Bumpy Ledge | Natural | Pavement & rubble | 0.1 | 0.6 | 29.3 | 22.5 | 4.1 | Long Bay | 12/2013, 6/2014, 9/2014, 12/2014 |
| Hammerhead Ledge | Natural | Pavement & rubble | 0.1 | 0.6 | 25.4 | 21.8 | 3.7 | Long Bay | 12/2013, 6/2014, 9/2014, 12/2014 |
| Lightning Bolt Ledge | Natural | Pavement & rubble | 0.2 | 0.8 | 28.5 | 21.8 | 8.8 | Long Bay | 12/2013, 6/2014, 9/2014, 12/2014 |
| Thumb Ledge | Natural | Pavement & rubble | 0.2 | 0.8 | 26.5 | 26.5 | 3.9 | Long Bay | 9/2013, 6/2014, 9/2014 |
| 210 Rock | Natural | Pavement & rubble | 0.2 | 0.9 | 30.2 | 25.2 | 8.2 | Onslow Bay | 9/2013, 6/2014, 10/2014 |
| Station Rock | Natural | Pavement & rubble | 0.3 | 1.1 | 15.6 | 23.4 | 10.1 | Onslow Bay | 9/2013, 10/2013, 6/2014, 8/2014, 10/2014, 5/2015 |
| West Rock | Natural | Pavement & rubble | 0.3 | 1.1 | 24.9 | 25.2 | 8.9 | Onslow Bay | 9/2013, 6/2014, 10/2014, 5/2015 |
| Southwest of Knuckle Buoy | Natural | Pavement & rubble | 0.3 | 1.4 | 14.3 | 23.9 | 7.0 | Onslow Bay | 8/2013, 10/2013, 8/2014, 10/2014, 5/2015 |
| Dallas Rocks | Natural | Pavement & rubble | 0.3 | 1.5 | 16.5 | 24.7 | 1.7 | Onslow Bay | 9/2013, 7/2014, 9/2014, 11/2014 |
| 23 Mile Ledge | Natural | Ledge | 0.4 | 1.6 | 28.7 | 25.5 | 3.3 | Onslow Bay | 6/2014, 9/2014 |
| 200 / 200 Ledge | Natural | Ledge | 0.4 | 1.5 | 25.3 | 25.0 | 1.6 | Onslow Bay | 9/2013, 9/2014, 11/2014 |
| Keypost Rock | Natural | Ledge | 0.4 | 1.5 | 15.0 | 25.0 | 8.1 | Onslow Bay | 9/2013, 7/2014, 10/2014, 10/2014 |
| 5 Mile Ledge | Natural | Ledge | 0.4 | 1.7 | 15.8 | 24.7 | 0.7 | Onslow Bay | 9/2013, 7/2014, 9/2014, 11/2014 |
| Northwest Reef | Natural | Ledge | 0.6 | 2.3 | 20.9 | 23.9 | 3.8 | Onslow Bay | 8/2013, 6/2014, 10/2014, 5/2015 |
| Barge Rock | Natural | Ledge | 0.6 | 2.2 | 16.1 | 24.8 | 6.6 | Onslow Bay | 8/2013, 7/2014, 9/2014, 10/2014 |
| 10 Fathom | Natural | Ledge | 0.6 | 2.4 | 20.9 | 23.7 | 3.8 | Onslow Bay | 8/2013, 10/2013, 6/2014, 8/2014, 10/2014, 5/2015 |
| Concrete Pipes, 2006 (AR-345) | Artificial | Concrete | 0.2 | 1.2 | 18.7 | 22.9 | --- | Onslow Bay | 7/2013, 10/2013, 6/2014, 10/2014, 10/2014, 5/2015 |
| Concrete Pipes, 2007 (AR-342) | Artificial | Concrete | 0.4 | 1.6 | 16.1 | 24.2 | --- | Onslow Bay | 9/2013, 6/2014, 8/2014, 10/2014, 5/2015 |
| Atlantic Beach Bridge (AR-320) | Artificial | Concrete | 0.7 | 2.9 | 15.2 | 23.3 | --- | Onslow Bay | 7/2013, 10/2013, 7/2014, 9/2014, 10/2014, 5/2015 |
| City of Houston | Artificial | Ship | 0.4 | 1.8 | 28.0 | 27.7 | --- | Long Bay | 9/2013, 6/2014, 9/2014 |
| Unknown Wreck | Artificial | Ship | 0.5 | 2.2 | 29.0 | 24.1 | --- | Long Bay | 12/20143, 6/2014, 9/2014 |
| Theodore Parker (AR-315) | Artificial | Ship | 0.5 | 2.4 | 10.2 | 24.3 | --- | Onslow Bay | 7/2013, 10/2013, 7/2014, 10/2014, 10/2014 |
| John D. Gill | Artificial | Ship | 0.7 | 3.1 | 25.1 | 25.0 | --- | Onslow Bay | 9/2013, 6/2014, 8/2014, 11/2014 |
| Cassimir | Artificial | Ship | 0.8 | 3.3 | 32.6 | 25.4 | --- | Onslow Bay | 9/2014, 11/2014 |
| Titan (AR-345) | Artificial | Ship | 0.8 | 3.5 | 16.6 | 23.0 | --- | Onslow Bay | 7/2013, 10/2013, 6/2014, 10/2014, 10/2014, 5/2015 |
| USS Indra (AR-342) | Artificial | Ship | 1.1 | 3.8 | 15.0 | 24.0 | --- | Onslow Bay | 6/2014, 8/2014, 5/2015 |
| Yard Oiler FS-26 (AR-300) | Artificial | Ship | 1.4 | 5.0 | 26.4 | 24.6 | --- | Onslow Bay | 6/2014 |
| Spar (AR-305) | Artificial | Ship | 1.4 | 5.1 | 28.3 | 25.1 | --- | Onslow Bay | 8/2013, 6/2014, 10/2014 |
| Raritan | Artificial | Ship | 1.7 | 5.7 | 21.5 | 23.3 | --- | Long Bay | 9/2013, 6/2014, 9/2014, 12/2014 |
| Alexander Ramsey (AR-370) | Artificial | Ship | 2.3 | 6.6 | 12.0 | 24.7 | --- | Onslow Bay | 9/2013, 7/2014, 9/2014, 11/2014 |
