# Supplementary material for: Flat and complex temperate reefs provide similar support for fish: Evidence for a unimodal species-habitat relationship
Source: PLoS One. 2017 Sep 5;12(9):e0183906. doi: 10.1371/journal.pone.0183906 (PMC5584758; doi:10.1371/journal.pone.0183906)
Supplement: S2 Table — Bold text indicates fishes in the federally managed snapper-grouper complex. Abundance values indicate the total numbers of each species observed across the 246 transects, as well as the number of each species observed on natural and artificial reefs. (DOCX) [file pone.0183906.s003.docx]

**S2 Table: Species list from 246 fish belt-transects conducted on warm-temperate reefs of the NC continental shelf.** Bold text indicates fishes in the federally managed snapper-grouper complex. Abundance values indicate the total numbers of each species observed across the 246 transects, as well as the number of each species observed on natural and artificial reefs.

| **Family** | **Genus** | **species** | **Common Name** | **Total Abundance** | **Natural**  **Abundance** | **Artificial**  **Abundance** |
| --- | --- | --- | --- | --- | --- | --- |
| Acanthuridae | *Acanthurus* | *chirurgus* | Doctorfish | 31 | 23 | 8 |
| Acanthuridae | *Acanthurus* | *coeruleus* | Blue Tang | 1 | 0 | 1 |
| Anguillidae | *Anguilla* | *rostrata* | American Eel | 2 | 2 | 0 |
| Apogonidae | *Apogon* | *pseudomaculatus* | Two Spot Cardinalfish | 85 | 75 | 10 |
| Apogonidae | *Apogon* | *planifrons* | Pale Cardinalfish | 2 | 2 | 0 |
| Atherinopsidae | *Menidia* | *menidia* | Silversides | 24,775 | 10,520 | 14,255 |
| **Balistidae** | ***Balistes*** | ***capriscus*** | **Grey Triggerfish** | **62** | **53** | **9** |
| Batrachoididae | *Opsanus* | *tau* | Oyster Toadfish | 48 | 31 | 17 |
| Belonidae | *Ablennes* | *hians* | Flat Needlefish | 3 | 3 | 0 |
| Blenniidae | *Parablennius* | *marmoreus* | Seaweed Blenny | 461 | 284 | 177 |
| Blenniidae | *Blenniidae* | sp. | Unknown Blenny | 110 | 22 | 88 |
| Blenniidae | *Hypleurochilus* | *geminatus* | Crested Blenny | 18 | 0 | 18 |
| Blenniidae | *Ophioblennius* | *macclurei* | Redlip Blenny | 1 | 1 | 0 |
| Carangidae | *Decapterus* | *macarellus* | Mackeral Scad | 56,904 | 20,038 | 36,866 |
| Carangidae | *Decapterus* | *punctatus* | Round Scad | 30,139 | 5,883 | 24,256 |
| Carangidae | *Selar* | *crumenophthalmus* | Big Eye Scad | 9,312 | 52 | 9,260 |
| Carangidae | *Decapterus* | sp. | Decapterus Species | 1,100 | 0 | 1,100 |
| **Carangidae** | ***Seriola*** | ***dumerili*** | **Greater Amberjack** | **763** | **123** | **640** |
| Carangidae | *Caranx* | *crysos* | Blue Runner | 257 | 225 | 32 |
| Carangidae | *Carangoides* | *bartholomaei* | Yellow Jack | 237 | 72 | 165 |
| **Carangidae** | ***Seriola*** | ***rivoliana*** | **Almaco Jack** | **97** | **13** | **84** |
| **Carangidae** | ***Caranx*** | ***ruber*** | **Bar Jack** | **62** | **7** | **55** |
| Carcharhinidae | *Carcharhinus* | *plumbeus* | Sandbar Shark | 1 | 0 | 1 |
| Chaetodontidae | *Chaetodon* | *ocellatus* | Spotfin Butterflyfish | 23 | 15 | 8 |
| Chaetodontidae | *Chaetodon* | *sedentarius* | Reef Butterflyfish | 11 | 8 | 3 |
| Cheloniidae | *Caretta* | *caretta* | Loggerhead Turtle | 1 | 1 | 0 |
| Cheloniidae | *Cheloniidae* | sp. | Unknown Turtle | 1 | 1 | 0 |
| Dasyatidae | *Dasyatis* | *americana* | Southern Stingray | 8 | 0 | 8 |
| Diodontidae | *Chilomycterus* | *schoepfi* | Striped Burrfish | 6 | 3 | 3 |
| Diodontidae | *Chilomycterus* | *antennatus* | Bridled Burrfish | 2 | 0 | 2 |
| Echeneidae | *Remora* | *remora* | Remora | 8 | 3 | 5 |
| **Ephippidae** | ***Chaetodipterus*** | ***faber*** | **Atlantic Spadefish** | **832** | **338** | **494** |
| Gobiidae | *Coryphopterus* | *eidolon* | Pallid Goby | 72 | 47 | 25 |
| Gobiidae | *Coryphopterus* | *glaucofraenum* | Bridled Goby | 63 | 36 | 27 |
| Gobiidae | *Gobiidae* | sp. | Unknown Goby | 54 | 24 | 30 |
| Gobiidae | *Gnatholepsis* | *thompsoni* | Goldspot Goby | 2 | 2 | 0 |
| Grammatidae | *Gramma* | *loreto* | Fairy Basslet | 2 | 2 | 0 |
| **Haemulidae** | ***Haemulon*** | ***aurolineatum*** | **Tomtate** | **118,724** | **26,161** | **92,563** |
| Haemulidae | *Haemulidae* | sp. | Unknown Juvenile Grunt | 37,773 | 14,465 | 23,308 |
| Haemulidae | *Orthopristis* | *chrysoptera* | Pigfish | 1,477 | 1,340 | 137 |
| **Haemulidae** | ***Haemulon*** | ***plumieri*** | **White Grunt** | **600** | **476** | **124** |
| Haemulidae | *Anisotremus* | *surinamensis* | Black Margate | 28 | 9 | 19 |
| **Haemulidae** | ***Haemulon*** | ***album*** | **White Margate** | **1** | 1 | 0 |
| Kyphosidae | *Kyphosus* | *sectatrix* | Bermuda Chub | 3 | 2 | 1 |
| Labridae | *Halichoeres* | *bivittatus* | Slippery Dick | 2,901 | 2,217 | 684 |
| Labridae | *Halichoeres* | *caudalis* | Painted Wrasse | 172 | 154 | 18 |
| Labridae | *Tautoga* | *onitis* | Tautog | 97 | 70 | 27 |
| Labridae | *Thalassoma* | *bifasciatum* | Bluehead Wrasse | 54 | 15 | 39 |
| Labridae | *Bodianus* | *rufus* | Spanish Hogfish | 27 | 4 | 23 |
| Labridae | *Halichoeres* | *radiatus* | Pudding Wife | 21 | 10 | 11 |
| Labridae | *Labridae* | sp. | Unknown Wrasse | 15 | 0 | 15 |
| **Labridae** | ***Lachnolaimus*** | ***maximus*** | **Hogfish** | **5** | **0** | **5** |
| Labridae | *Doratonotus* | *megalepis* | Dwarf Wrasse | 2 | 2 | 0 |
| Lotidae | *Brosme* | *brosme* | Cusk | 1 | 0 | 1 |
| **Lutjanidae** | ***Rhomboplites*** | ***aurorubens*** | **Vermillion Snapper** | **18,764** | **193** | **18,571** |
| **Lutjanidae** | ***Lutjanus*** | ***campechanus*** | **Red Snapper** | **30** | **22** | **8** |
| **Lutjanidae** | ***Lutjanus*** | ***mahogoni*** | **Mahogany Snapper** | **27** | **17** | **10** |
| **Lutjanidae** | ***Lutjanus*** | ***synagris*** | **Lane Snapper** | **6** | **2** | **4** |
| **Lutjanidae** | ***Lutjanus*** | ***griseus*** | **Gray Snapper** | **5** | **0** | **5** |
| **Lutjanidae** | ***Ocyurus*** | ***chrysurus*** | **Yellowtail Snapper** | **1** | **1** | **0** |
| Monacanthidae | *Stephanolepis* | *hispidus* | Planehead Filefish | 55 | 27 | 28 |
| Mullidae | *Mullus* | *auratus* | Red Goatfish | 107 | 2 | 105 |
| Mullidae | *Pseudupeneus* | *maculatus* | Spotted Goatfish | 56 | 49 | 7 |
| Mullidae | *Upeneus* | *parvus* | Dwarf Goatfish | 42 | 8 | 34 |
| Mullidae | *Mulloidichthys* | *martinicus* | Yellow Goatfish | 37 | 7 | 30 |
| Muraenidae | *Muraena* | *retifera* | Reticulate Moray | 12 | 10 | 2 |
| Muraenidae | *Gymnothorax* | *milaris* | Goldentail Moray | 4 | 3 | 1 |
| Muraenidae | *Anguilliformes* | sp. | Unknown Eel | 1 | 1 | 0 |
| Odontaspididae | *Carcharias* | *taurus* | Sandtiger Shark | 49 | 1 | 48 |
| Ophichthidae | *Myrichthys* | *ocellatus* | Goldspotted Eel | 2 | 0 | 2 |
| Osteichthyes | *Osteichthyes* | sp. | Unknown Fish Species | 22 | 18 | 4 |
| Ostraciidae | *Acanthostracion* | *polygonius* | Honeycomb Cowfish | 1 | 1 | 0 |
| Ostraciidae | *Acanthostracion* | *quadricornis* | Scrawled Cowfish | 1 | 0 | 1 |
| Ostraciidae | *Rhinesomus* | *triqueter* | Smooth Trunkfish | 1 | 0 | 1 |
| Paralichthyidae | *Paralichthys* | *albigutta* | Gulf Flounder | 36 | 25 | 11 |
| Paralichthyidae | *Paralichthys* | *dentatus* | Summer Flounder | 22 | 9 | 13 |
| Paralichthyidae | *Paralichthys* | *lethostigma* | Southern Flounder | 5 | 1 | 4 |
| Phycidae | *Urophycis* | *earllii* | Carolina Hake | 22 | 17 | 5 |
| Pomacanthidae | *Holacanthus* | *bermudensis* | Blue Angelfish | 138 | 73 | 65 |
| Pomacanthidae | *Holacanthus* | *ciliaris* | Queen Angelfish | 9 | 4 | 5 |
| Pomacanthidae | *Pomacanthidae* | sp. | Unknown Juvenile Angelfish | 3 | 3 | 0 |
| Pomacentridae | *Chromis* | *scotti* | Purple Reef Fish | 1,022 | 60 | 962 |
| Pomacentridae | *Pomacentridae* | sp. | Unknown Juvenile Damselfish | 127 | 101 | 26 |
| Pomacentridae | *Stegastes* | *leucostictus* | Beaugregory Damselfish | 105 | 26 | 78 |
| Pomacentridae | *Stegastes* | *variabilis* | Cocoa Damselfish | 82 | 69 | 13 |
| Pomacentridae | *Stegastes* | *partitus* | Bicolor Damselfish | 71 | 42 | 29 |
| Pomacentridae | *Chromis* | *cyanea* | Blue Chromis | 23 | 0 | 23 |
| Pomacentridae | *Stegastes* | *diencaeus* | Longfin Damselfish | 9 | 0 | 9 |
| Pomacentridae | *Stegastes* | *adustus* | Dusky Damselfish | 3 | 0 | 3 |
| Pomacentridae | *Abudefduf* | *saxatilis* | Sergeant major | 2 | 1 | 1 |
| Pomacentridae | *Chromis* | *enchrysura* | Yellowtail Reef Fish | 2 | 2 | 0 |
| Pomacentridae | *Abudefduf* | *taurus* | Night Sergeant | 1 | 1 | 0 |
| Ptereleotridae | *Ptereleotris* | *calliura* | Blue Dartfish | 27 | 27 | 0 |
| Rachycentridae | *Rachycentron* | *canadum* | Cobia | 1 | 0 | 1 |
| Rajidae | *Dipturus* | *laevis* | Barndoor Skate | 1 | 1 | 0 |
| Rhincodontidae | *Ginglymostoma* | *cirratum* | Nurse Shark | 1 | 0 | 1 |
| Scaridae | *Sparisoma* | *atomarium* | Green Blotch Parrotfish | 10 | 8 | 2 |
| Scaridae | *Scarus* | *iseri* | Striped Parrotfish | 3 | 3 | 0 |
| Sciaenidae | *Pareques* | *umbrosus* | Cubbyu | 1,332 | 715 | 617 |
| Sciaenidae | *Pareques* | *acuminatus* | High Hat | 6 | 3 | 3 |
| Scombridae | *Scomberomorus* | *maculatus* | Spanish Mackerel | 594 | 2 | 592 |
| Scombridae | *Scomberomorus* | *cavalla* | King Mackerel | 250 | 0 | 250 |
| Scombridae | *Euthynnus* | *alletteratus* | Little Tunny | 154 | 8 | 146 |
| Scorpaenidae | *Pterois* | *volitans* | Lionfish | 47 | 12 | 35 |
| Scorpaenidae | *Scorpaena* | *plumieri* | Spotted Scorpionfish | 3 | 2 | 1 |
| **Serranidae** | ***Centropristis*** | ***striata*** | **Black Sea Bass** | **3,208** | **2,283** | **925** |
| Serranidae | *Serranus* | *subligarius* | Belted Sandfish | 1,228 | 731 | 497 |
| **Serranidae** | ***Mycteroperca*** | ***microlepis*** | **Gag** | **390** | **197** | **193** |
| **Serranidae** | ***Centropristis*** | ***ocyurus*** | **Bank Sea Bass** | **245** | **200** | **45** |
| **Serranidae** | ***Mycteroperca*** | ***phenax*** | **Scamp** | **199** | **108** | **91** |
| Serranidae | *Rypticus* | *maculatus* | White Spotted Soapfish | 118 | 58 | 60 |
| Serranidae | *Diplectrum* | *formosum* | Sand Perch | 39 | 34 | 5 |
| **Serranidae** | ***Epinephelus*** | ***guttatus*** | **Red Hind** | **2** | **0** | **2** |
| Serranidae | *Hypoplectrus* | *puella* | Barred Hamlet | 2 | 2 | 0 |
| Serranidae | *Serranus* | *baldwini* | Lantern Bass | 2 | 2 | 0 |
| Serranidae | *Serranus* | *phoebe* | Tattler Bass | 2 | 2 | 0 |
| Serranidae | *Serranus* | *tigrinus* | Harlequin Bass | 2 | 2 | 0 |
| Serranidae | *Liopropoma* | *eukrines* | Wrasse Basslet | 1 | 1 | 0 |
| **Serranidae** | ***Mycteroperca*** | ***interstitialis*** | **Yellowmouth Grouper** | **1** | 0 | 1 |
| Serranidae | *Rypticus* | *saponaceus* | Greater Soapfish | 1 | 0 | 1 |
| Sparidae | *Diplodus* | *holbrookii* | Spottail Pinfish | 16,207 | 6,661 | 9,546 |
| Sparidae | *Stenotomus* | *chrysops* | Scup | 1010 | 475 | 535 |
| Sparidae | *Diplodus* | *argenteus* | Silver Porgy | 590 | 9 | 581 |
| Sparidae | *Archosargus* | *probatocephalus* | Sheepshead | 518 | 146 | 372 |
| Sparidae | *Lagodon* | *rhomboides* | Pinfish | 275 | 83 | 192 |
| **Sparidae** | ***Stenotomus*** | ***caprinus*** | **Longspine Porgy** | **245** | **189** | **56** |
| Sparidae | *Calamus* | *penna* | Sheepshead Porgy | 240 | 164 | 76 |
| Sparidae | *Calamus* | *proridens* | Littlehead Porgy | 126 | 124 | 2 |
| **Sparidae** | ***Calamus*** | ***calamus*** | **Saucereye Porgy** | **117** | **71** | **46** |
| Sparidae | *Sparidae* | sp. | Unknown Juvenile Porgy | 100 | 30 | 70 |
| **Sparidae** | ***Calamus*** | ***nodosus*** | **Knobbed Porgy** | **28** | **26** | **2** |
| **Sparidae** | ***Calamus*** | ***bajonado*** | **Jolthead Porgy** | **15** | **10** | **5** |
| **Sparidae** | ***Sparidae*** | **sp.** | **Unknown Porgy** | **10** | **6** | **4** |
| Sparidae | *Archosargus* | *rhomboidalis* | Sea Bream | 4 | 0 | 4 |
| **Sparidae** | ***Pagrus*** | ***pagrus*** | **Red Porgy** | **3** | **0** | **3** |
| Sphyraenidae | *Sphyraena* | *guachancho* | Guaguanche | 790 | 200 | 590 |
| Sphyraenidae | *Sphyraena* | *barracuda* | Barracuda | 80 | 7 | 73 |
| Sphyraenidae | *Sphyraena* | *borealis* | Northern Sennet | 1 | 1 | 0 |
| Synodontidae | *Synodus* | *foetens* | Inshore Lizardfish | 4 | 3 | 1 |
| Tetraodontidae | *Canthigaster* | *rostrata* | Sharpnose Puffer | 56 | 23 | 33 |
| Tetraodontidae | *Sphoeroides* | *spengleri* | Bandtail Puffer | 21 | 11 | 10 |
