# Supplementary material for: Flat and complex temperate reefs provide similar support for fish: Evidence for a unimodal species-habitat relationship
Source: PLoS One. 2017 Sep 5;12(9):e0183906. doi: 10.1371/journal.pone.0183906 (PMC5584758; doi:10.1371/journal.pone.0183906)
Supplement: S3 Table — Environmental variables include digital reef rugosity (DRR (m)), squared digital reef rugosity (DRR 2 (m)), average reef depth (m), average water temperature (°C), and standard deviation of sediment cover (m) approximating sediment dynamics. Coefficients, standard error (SE), Z-values and P-values are provided for each environmental parameter. Bold values indicate significance. Interpretation of the pattern (unimodal, linear, or non-significant (NS)) between rugosity and the fish abundance are displayed for each model. Model results displayed here were from the best models that we evaluated. (DOCX) [file pone.0183906.s004.docx]

**S3 Table: GLM results for the relationship between fish abundance and environmental predictor variables by reef type and fish size class.** Environmental variables include digital reef rugosity (DRR (m)), squared digital reef rugosity (DRR ^2^ (m)), average reef depth (m), average water temperature (^o^C), and standard deviation of sediment cover (m) approximating sediment dynamics. Coefficients, standard error (SE), Z-values and P-values are provided for each environmental parameter. Bold values indicate significance. Interpretation of the pattern (unimodal, linear, or non-significant (NS)) between rugosity and the fish abundance are displayed for each model. Model results displayed here were from the best models that we evaluated.

| **Reef type** | **Size class** | **Predictor variable** | **Coefficient** | **Standard error** | **z-value** | **P-value** |
| --- | --- | --- | --- | --- | --- | --- |
| Natural | Small | Intercept | -1.84 | 1.32 | -1.39 | 0.16 |
|  |  | **DRR** | 10.29 | 2.79 | 3.68 | **0.0002** |
|  |  | **DRR^2^** | -11.08 | 3.06 | -3.62 | **0.0003** |
|  |  | **Depth** | 0.12 | 0.03 | 4.34 | **<0.0001** |
|  |  | **Temperature** | 0.15 | 0.05 | 3.22 | **0.001** |
|  |  | **Sediment** | -0.08 | 0.03 | -2.60 | **0.01** |
| Artificial | Small | Intercept | 4.01 | 0.54 | 7.38 | <0.0001 |
|  |  | DRR | 1.21 | 0.78 | 1.55 | 0.12 |
|  |  | **DRR^2^** | -0.34 | 0.27 | -1.27 | 0.20 |
|  |  | **Depth** | 0.11 | 0.25 | 4.38 | **<0.0001** |
| Natural | Medium | Intercept | 4.15 | 0.88 | 4.71 | <0.0001 |
|  |  | DRR | 2.04 | 2.67 | 0.76 | 0.45 |
|  |  | **DRR^2^** | -1.99 | 2.94 | -0.68 | 0.50 |
|  |  | **Depth** | 0.05 | 0.03 | 1.94 | **0.05** |
| Artificial | Medium | Intercept | 5.39 | 1.25 | 4.31 | <0.0001 |
|  |  | DRR | 1.09 | 0.72 | 1.51 | 0.13 |
|  |  | **DRR^2^** | -0.37 | 0.24 | -1.50 | 0.13 |
|  |  | **Depth** | 0.16 | 0.02 | 6.90 | **<0.0001** |
|  |  | **Temperature** | -0.11 | 0.05 | -2.28 | **0.02** |
| Natural | Large | Intercept | 1.75 | 0.28 | 6.17 | <0.0001 |
|  |  | DRR | 0.34 | 0.56 | 0.61 | 0.54 |
|  |  | **Sediment** | 0.07 | 0.03 | 2.48 | **0.01** |
| Artificial | Large | Intercept | 0.59 | 0.62 | 0.95 | 0.34 |
|  |  | **DRR** | 0.80 | 0.23 | 3.55 | **0.0003** |
|  |  | **Depth** | 0.08 | 0.03 | 2.71 | **0.007** |
| Natural | Extra large | Intercept | -1.65 | 0.75 | -2.20 | 0.03 |
|  |  | **DRR** | 1.30 | 0.68 | 1.92 | **0.06** |
|  |  | **Depth** | 0.09 | 0.03 | 3.44 | **0.001** |
| Artificial | Extra large | Intercept | -0.49 | 0.74 | -0.67 | 0.51 |
|  |  | **DRR** | 2.34 | 1.09 | 2.15 | **0.03** |
|  |  | **DRR^2^** | -0.82 | 0.38 | -2.17 | **0.03** |
|  |  | **Depth** | 0.10 | 0.03 | 3.09 | **0.002** |
